# RAB5A regulates cell proliferation and lipid metabolism by modulating mitochondrial ROS via AMPK signaling pathway in ovarian granulosa cells

**DOI:** 10.1016/j.redox.2026.104183

**Published:** 2026-04-29

**Authors:** Shao-Hong Liu, Ping Yang, Bing-Hong Zhu, Hong-Yu Li, Shan Wang, Yong Wang, Xiao-Man Liu

**Affiliations:** a Department of Clinical Laboratory, Shandong Provincial Hospital Affiliated to Shandong First Medical University, Jinan, 250021, China; b Central Laboratory, Shandong Provincial Hospital Affiliated to Shandong First Medical University, Jinan, 250021, China; c Department of Obstetrics and Gynecology, Shandong Provincial Hospital Affiliated to Shandong First Medical University, Jinan, 250021, China; d Department of Obstetrics and Gynecology, The First Affiliated Hospital of Shandong First Medical Universit, Shandong Provincial Qianfoshan Hospital, Jinan, 250014, China; e Department of Reproductive Medicine, Department of Obstetrics and Gynecology, Shandong Provincial Hospital Affiliated to Shandong First Medical University, Jinan, Shandong, 250021, China; f Shandong Key Laboratory of Reproductive Research and Birth Defect Prevention, Shandong First Medical University, Jinan, Shandong, 250021, China; g Jinan Engineering Laboratory of Reproductive Diagnosis and Treatment Technology, Shandong Provincial Hospital Affiliated to Shandong First Medical University, Jinan, Shandong, 250021, China; h Shandong Second Medical University, Weifang, Shandong, 252422, China

**Keywords:** RAB5A, Mitochondria, AMPK, MIGA2, Lipid metabolism, Cell proliferation

## Abstract

Ovarian granulosa cells (GCs) play a crucial role in follicle development and hormone production. These functions require substantial energy, supported by mitochondrial activity and balanced lipid metabolism. RAB5 is known to maintain mitochondrial homeostasis; however, its role in regulating lipid metabolism via the energy-sensing AMP-activated protein kinase (AMPK) pathway remains unclear. In polycystic ovary syndrome (PCOS), a disorder often linked to metabolic and mitochondrial defects in GCs, RAB5A levels are significantly reduced in obese subtypes. In this study, we demonstrate that RAB5A deficiency disrupts lipid metabolism and impairs normal cell proliferation, characterized by increased mitochondrial stress (increased reactive oxygen species) and activation of mitophagy via AMPK. RAB5A may coordinate with MIGA2, a protein involved in regulating mitochondria–lipid droplet interactions, to modulate lipid metabolism via AMPK activity. Notably, activating AMPK with AICAR reverses the adverse effects of RAB5A loss. Collectively, these findings identify RAB5A as a key regulator of GC function and a potential therapeutic target in obese PCOS.

## Highlights

• RAB5 expression is increased in luteinized granulosa cells, its expression is reduced in growing follicles and corpus luteum of PCOS ovaries.
• RAB5A but not RAB5B is essential for granulosa cell proliferation and its deficiency leads to increased ROS and excessive mitophagy.
• RAB5A and MIGA2 reciprocally regulate each other and may maintain phospholipids homeostasis via AMPK signaling.
• Activating the AMPK pathway with the AICAR can counteract the damaging effects of RAB5A deficiency.

## Graphical abstract

Graphical abstract for this study. In the obese PCOS model, follicular granulosa cells exhibit decreased RAB5A expression. Reduction of RAB5A expression triggers: Mitochondrial Dysfunction: Increased mitochondrial ROS and decreased ATP, leading to mitochondrial damage and PINK1/Parkin-mediated mitophagy. Lipid Metabolism Disorder: Reduced levels of glycerophospholipids, sphingolipids, and glycerolipids. Disrupted Lipid Trafficking/Synthesis: Downregulation of the mitochondrial outer membrane protein MIGA2, a key factor in lipid transfer and synthesis, further contributes to the metabolic disorder. These effects converge on the suppression of AMPK signaling, which is inhibited by either RAB5A or MIGA2 knockdown. Crucially, the AMPK activator AICAR rescues the high ROS levels, cell proliferation defect and the lipid imbalance in KGN cells. Targeting this pathway with AICAR represents a potential therapeutic strategy for obese PCOS.

**Figure:**
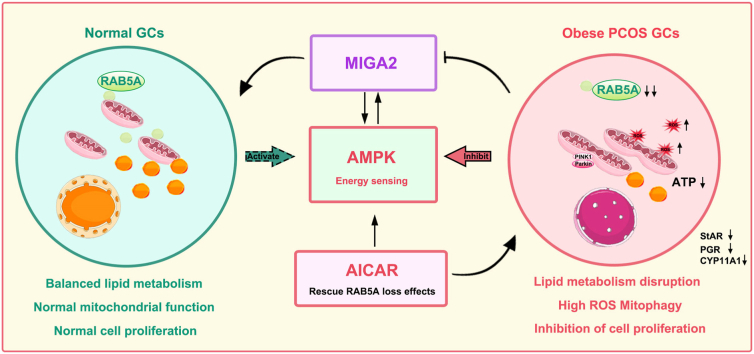

## 1 Introduction

Follicular granulosa cells (GCs) are essential for follicle development and ovarian endocrine function. Their proliferation, differentiation, and steroidogenic activity directly regulate follicular development. Lipid metabolism provides energy, maintains membrane structures, and shapes the follicular microenvironment, which is essential for supporting GC functions [1]. Cholesterol, a versatile lipid, serves as the precursor for steroid hormone biosynthesis [2]. Mitochondria act as the primary site of lipid metabolism, facilitating processes such as ketone body synthesis and fatty acid oxidation. Their functional integrity directly impacts lipid utilization efficiency and steroidogenic capacity in GCs [3,4]. Consequently, elucidating the regulatory mechanisms underlying mitochondrial function and lipid metabolism in GCs is critical for understanding ovarian metabolic homeostasis.

Small guanosine triphosphate (GTP)-binding proteins of the Ras-related in brain (Rab) family regulate intracellular membrane trafficking by controlling the biosynthesis, transport, tethering, and fusion of membrane-bound organelles and vesicles [5]. RAB5, a classical early endosome marker, comprises three isoforms: RAB5A, RAB5B, and RAB5C [6]. This marker regulates key vesicular processes such as vesicle budding and fusion [7], and also participates in autophagy and energy metabolism [8,9]. Moreover, its effector protein Ferritin heavy chain-mediated iron transport for erythroid maturation (FERRY) helps maintain mitochondrial homeostasis by binding to mRNAs encoding mitochondrial proteins and mediating their localization to early endosomes [10]. These findings suggest that RAB5 may regulate mitochondrial function; however, its role in lipid metabolism within GCs remains unclear.

The cellular energy sensor adenosine monophosphate (AMP)-activated protein kinase (AMPK) serves as a key signaling node linking mitochondrial function to metabolic regulation. AMPK activation promotes mitochondrial biogenesis and regulates mitochondrial function during processes such as oocyte aging [11]. AMPK also directly influences lipid metabolism by facilitating lipid droplet-mitochondria crosstalk via Rab8a to enhance fatty acid β-oxidation [12]. Ultimately, mitochondrial activity can regulate AMPK activity and influence cellular metabolism through Hippo/Yes-associated protein (YAP) pathways [13,14], forming a regulatory loop of “mitochondrial function-AMPK signaling-metabolic output". However, whether RAB5 modulates mitochondrial function via AMPK to regulate lipid metabolism in GCs remains unexplored.

Polycystic ovary syndrome (PCOS) is a common disorder affecting 10%–18% of reproductive-aged women and is characterized by reproductive and metabolic disturbances. Notably, 60%–70% of patients with PCOS are overweight or obese [15]. A key pathological feature is dysregulated lipid metabolism coupled with mitochondrial dysfunction in GCs. Elevated free fatty acids (FFA) in the follicular fluid of patients with PCOS can induce mitochondrial abnormalities in GCs, thereby suppressing cell proliferation, promoting apoptosis, and ultimately impairing follicular development [1,16,17]. Previous studies have reported significantly decreased RAB5A mRNA levels in luteinized GCs from obese patients with PCOS [18]. However, the relationship between decreased RAB5A expression and mitochondrial dysfunction, as well as lipid metabolic defects in GCs, remains unclear.

This study demonstrates that RAB5 regulates mitochondrial reactive oxygen species (ROS) via the AMPK signaling pathway and collaborates with Mitoguardin-2 (MIGA2) to modulate lipid metabolism in follicular GCs. These findings expand the theoretical framework of coordination among vesicular trafficking, signaling pathways, and organelle functions. Moreover, they provide new insights into the pathological mechanisms and potential therapeutic targets for reproductive disorders characterized by GC metabolic dysfunction, including PCOS.

## 2 Materials and methods

### 2.1 Cell culture and treatments

The human ovarian granulosa cell tumor cell line KGN (RIKEN BioResource Center, IBR, Japan) was cultured in DMEM/F-12 medium (Gibco, USA) supplemented with 10% fetal bovine serum (FBS; Gibco, USA) and 1% antibiotics (100 IU/mL penicillin and 100 μg/mL streptomycin) (Gibco, USA). HeLa cells (National Collection of Authenticated Cell Cultures, China) were cultured in DMEM medium (Gibco, USA) supplemented with 10% FBS and 1% penicillin-streptomycin. All cells were incubated in a humid environment at 37 °C with 5% CO_2_. To stimulate cyclic adenosine monophosphate (cAMP) production and induce luteinization, KGN cells were treated with forskolin (FSK; 10 μM) and phorbol 12-myristate 13-acetate (PMA; 20 nM) (Sigma, USA) for 24 h. To trigger mitochondrial stress or induce mitophagy, cells were treated with 10 μM Carbonyl Cyanide3-ChloroPhenylhydrazone (CCCP; Sigma, USA) for 4 h. Autophagy was induced by starvation in Hank's Balanced Salt Solution (HBSS), and the process was arrested by chloroquine (CQ; MCE, USA) treatment for 4 h. The 5-Aminoimidazole-4-carboxamide ribonucleoside (AICAR; MCE, USA) was used to activate the AMPK signaling at the concentration of 0.5 mM for 16 h.

### 2.2 Dehydroepiandrosterone (DHEA)-induced PCOS mouse model

All mice used were specific pathogen-free (SPF)-grade and purchased from Beijing Vital River Laboratory Animal Technology Co., Ltd. The mice were housed in the Animal Facility of Shandong Provincial Hospital Affiliated to Shandong First Medical University, and all animal experiments were conducted according to the guidelines of the Animal Care & Welfare Committee of Shandong Provincial Hospital Affiliated to Shandong First Medical University. Female mice were housed with free access to food and water, under a 12-h light/dark cycle (lights on at 7:00 a.m. and off at 7:00 p.m.).

At the age of 4 weeks, female C57BL/6J mice were randomly divided into three groups after purchase: the control group with normal diet (Control), the control with high-fat diet (Control-HF) group and the DHEA with high-fat diet (DHEA-HF) group. The control groups were subcutaneously injected with sesame oil at the nape of the neck. The experimental group received subcutaneous injection of DHEA (MCE, USA) dissolved in sesame oil using the same method. For DHEA preparation, 6 mg of DHEA was dissolved in 0.10 mL of sesame oil per 100 g of mouse body weight. Both groups were administered 0.10 mL of the corresponding solution once daily for 21 consecutive days. Ovaries and the serum were collected for verification and detection. The serum testosterone was measured by chemiluminescence (Roche Diagnostics, Germany). The glucose tolerance test was performed on the 22nd day after the completion of the construction.

### 2.3 RNA interference, lentiviral constructs and plasmid constructions

The small interfering RNAs (siRNAs) were designed to target specific genes for knockdown to assess their cellular functions. The targeting sequences used were as follows: homo-RAB5A siRNA: GGAAGAGGAGUAGACCUUATT; homo-RAB5B siRNA: GACAGCUAUGAACGUGAAUTT. KGN or HeLa cells were transfected with the corresponding siRNAs using lipofectamine RNAiMax (Invitrogen, USA) at a final concentration of 120 nM for 48 h. Lentiviral constructs and siRNAs targeting MIGA1 and MIGA2 were designed and applied as previously studies [19,20]. Plasmids expressing MIGA1-Green Fluorescent Protein (GFP), MIGA2-GFP, MIGA2-HA and MFN2-GFP were constructed according to established methods [21]. The RAB5A-GFP plasmid was generated by cloning the human RAB5A cDNA into the pEGFP-N1 vector, and its sequence was successfully verified by sequencing and detection. The mCh-Rab5 was a gift from Gia Voeltz (Addgene plasmid # 49201; http://n2t.net/addgene:49201; RRID:Addgene_49201) [22].

### 2.4 Quantitative real-time PCR (qRT-PCR or qPCR)

Total RNA was extracted from cells using TRIzol Reagent (Invitrogen, USA) and reverse-transcribed into cDNA using the PrimeScript RT reagent Kit with gDNA Eraser (TaKaRa, Japan). The qRT-PCR was performed in triplicate for each sample on a LightCycler 480 II instrument (Roche, Germany) using SYBR® Green PCR Master Mix (TaKaRa, Japan). Relative mRNA expression of target genes were calculated using the comparative crossing points (Cp) method and formula 2^−ΔΔCp^. Each experiment was repeated independently at least three times. The primers used in this study are listed in the supplementary files (Table S1).

### 2.5 Western blotting (WB)

Proteins samples were separated by sodium dodecyl sulfate-polyacrylamide gel electrophoresis (SDS-PAGE), and transferred onto polyvinylidene difluoride (PVDF) membranes. After blocking with 5% skim milk, the membranes were incubated with specific primary antibodies overnight at 4 °C, followed by incubation with horseradish peroxidase (HRP)-conjugated secondary antibodies. Protein bands were visualized using chemiluminescent HRP Substrates (Millipore, USA) and imaged with a GelDoc2 XR Gel Documentation System (BioRad, USA). Band intensities were quantified using ImageJ software, and each experiment was repeated at least three times independently. The antibodies used in this study are listed in the supplementary files (Table S2).

### 2.6 Co-immunoprecipitation (Co-IP) assay

To determine if MIGA2 and RAB5A physically interact, a co-IP assay was performed. Cells were co-transfected with plasmids expressing MIGA2 tagged with HA (MIGA2-HA) and RAB5A tagged with GFP (RAB5A-GFP). A positive interaction control (co-transfection of MIGA2-HA and MIGA2-GFP) was included. After transfection for 48 h, cells were lysed in RIPA lysis buffer to preserve potential protein-protein interactions. The lysate was then incubated with anti-HA antibody that was pre-bound to Protein A/G beads. This allowed for the immunoprecipitation of the “bait" protein (MIGA2-HA) and any associated “prey" proteins. Following several washes to remove non-specifically bound proteins, the bead-bound complexes were eluted by boiling in SDS-PAGE loading buffer. The eluted proteins were then separated by electrophoresis, transferred to a PVDF membrane, and analyzed by WB assay. The membrane was probed with an anti-GFP antibody to detect the presence of co-precipitated RAB5A-GFP.

### 2.7 Cell viability assay

Cell viability was measured using the Cell Counting Kit-8 (CCK-8) (Beyotime Biotechnology, China) according to the manufacturer's protocol. Briefly, cells were seeded in aliquots and seeded in a 96-well plate at a density of 1 × 10^4^ cells per well. Following treatment, CCK-8 reagent was added, and the absorbance at 450 nm was measured using a Multiskan Go microplate reader (BioTeK, USA). Each treatment group was assayed in triplicate wells, and each experiment was independently repeated three times.

### 2.8 5-Ethynyl-2′-deoxyuridine (EdU) assay

EdU assay was performed using EdU assay kit (RIBOBIOCo., China) according to the manufacturer's instructions. Briefly, KGN cells were seeded in 96-well plates at a density of 1 × 10^4^ cells/mL (100 μL per well). After treatments, EdU was added to the culture medium and incubated for 2 h before fixation and detection. Images were acquired using the ImageXpress Micro Confocal device (Molecular Devices, USA). The number of EdU-positive cells was quantified with ImageJ software, and the proliferation rate was calculated as the percentage of EdU-positive cells from a count of at least 500 cells per sample.

### 2.9 Live cell imaging

Mitochondrial morphology was visualized by staining cells with 250 nM Mitotracker Red (Invitrogen, USA), cellular ROS was detected by staining with the DCFH-DA probe in the ROS detection kit (Beyontime, China), while mitochondrial ROS was detected using 3 μM MitoSOX (Invitrogen, USA) according to the instruction. Lysosomes were identified with LysoTracker Green staining (Invitrogen, USA). Lipid droplet was visualized by staining with the Boron-Dipyrromethene (BODIPY) 493/503 (Invitrogen, USA). Live cells were incubated with these dyes individually or in combination for 30 min at 37 °C in the dark, using glass-bottom dishes. Following staining, nuclei were counterstained with Hoechst 33342 or not. Images were acquired using TCS SP8 confocal microscope (Leica, Germany). Mitochondrial morphology was categorized into four distinct types, the proportion of cells exhibiting each type was quantified. Fluorescence intensity and trait prevalence were quantified in ImageJ.

### 2.10 Immunofluorescence (IF)

Cells were fixed in 4% paraformaldehyde (PFA) for 30 min, permeabilized with 0.1% Triton X-100 in PBS for 10 min, and blocked with 5% bovine serum albumin (BSA) for 30 min. Subsequently, cells were incubated with primary antibodies overnight at 4 °C. After washing, cells were incubated with fluorophore-conjugated secondary antibodies for 30 min at room temperature in the dark. Nuclei were stained with DAPI, and fluorescence images were captured using a Leica TCS SP8 confocal microscope (Leica, Germany).

### 2.11 Immunohistochemistry (IHC)

Mouse ovaries were fixed in 4% PFA, embedded in paraffin, and sectioned. Ovary sections were incubated with primary antibodies, followed by incubation with a biotin-labeled secondary antibodies (VECTASTAIN ABC kit, Vector Laboratories, USA). Signal was developed using 3,3′-diaminobenzidine (DAB, Vector Laboratories, USA), and sections were counterstained with hematoxylin. Images were captured using the TissueFAXS Plus system (TissueGnostics, Austria).

### 2.12 Adenosine triphosphate (ATP) assay

Cellular ATP levels were measured using a luciferase-based assay. Briefly, cells were lysed, and the lysates were mixed with a reaction reagent containing luciferase and luciferin. The luminescence intensity, which is proportional to ATP concentration, was measured using an LB 960 microplate luminometer (Berthold Technologies, BW, Germany). A standard curve generated with known ATP concentrations was used to calculate sample ATP levels. Blank controls and standard samples were included in each assay to ensure reliability. Each treatment group was assayed in triplicate wells, and each experiment was independently repeated three times.

### 2.13 Metabolomics analysis

Cell samples were collected and divided into two groups, each with three replicates. After extraction, the samples were analyzed using a Liquid Chromatography-Electrospray Ionization-Tandem Mass Spectrometry (LC-ESI-MS/MS) system. The system consisted of an ultra-performance liquid chromatography (UPLC) unit (ExionLC AD) and a tandem mass spectrometer (QTRAP® system). For hydrophilic compounds, the analytical conditions were configured as follows, UPLC: column, Waters ACQUITY UPLC HSS T3 C18 (1.8 μm,2.1 mm∗100 mm); column temperature, 40 °C; flow rate, 0.4 mL/min; injection volume, 2 μL; solvent system, water (0.1% formic acid): acetonitrile (0.1% formic acid); gradient program, 95:5 V/V at 0 min, 10:90 V/V at 11.0 min,10:90 V/V at 12.0 min, 95:5 V/V at 12.1 min, 95:5 V/V at 14.0 min. For hydrophobic compounds, the analytical conditions were as follows, UPLC: column, Thermo Accucore™ C30 (2.6 μm, 2.1 mm∗100 mm i.d.); solvent system, A: acetonitrile/water (60/40,V/V, 0.1% formic acid, 10 mmol/L ammonium formate), B: acetonitrile/isopropanol (10/90 V/V, 0.1% formic acid, 10 mmol/L ammonium formate); gradient program, A/B 80:20, V/V at 0 min, 70:30 V/V at 2.0 min, 40:60 V/V at 4 min, 15:85 V/V at 9 min, 10:90 V/V at 14 min, 5:95 V/V at 15.5 min, 5:95 V/V at 17.3 min, 80:20 V/V at 17.3 min, 80:20 V/V at 20 min; flow rate, 0.35 ml/min; temperature, 45 °C; Injection volume: 2 μL. The effluent was alternatively connected to an ESI-triple quadrupole-linear ion trap (QTRAP)-MS.

Standardization using unit variance scaling (UV), also known as Z-score standardization, standardizes data based on the mean and SD of the original data. The processed data conforms to a standard normal distribution. Calculation method: After centering the original data using the formula: x – μ (μ is the mean), divide each value by the SD (σ) of the variable. The formula is: x′ = (x − μ)/σ. Multiple testing correction using the Benjamini-Hochberg FDR (q < 0.05). The coefficient of variation (CV) value is the ratio of the standard deviation (SD) of the raw data to its mean value. The Empirical Cumulative Distribution Function (ECDF) is used to analyze the frequency of substances with a CV below a reference value. In the data from this study, the proportion of substances with a CV below 0.3 in the quality control (QC) samples was higher than 85%, indicating that the experimental data is highly stable.

Differential metabolites were determined based on Variable Importance in Projection (VIP) values (VIP >1) and P-values (P-value <0.05, Student's t-test). The VIP values were extracted from the results of Orthogonal Partial Least Squares-Discriminant Analysis (OPLS-DA). Prior to the OPLS-DA, the data was log-transformed (log_2_) and mean-centered. To avoid overfitting, a permutation test (with 200 permutations) was conducted. Identified metabolites were annotated using the Kyoto Encyclopedia of Genes and Genomes (KEGG) Compound database. Pathways to which the significantly regulated metabolites were mapped were then subjected to Metabolite Set Enrichment Analysis (MSEA). The significance of these pathways was determined by the P-values from a hypergeometric test.

### 2.14 Statistical analysis

Statistical analysis and graphing were performed using GraphPad Prism 8.0 software. All data are presented as mean ± SD. Comparisons between two groups were analyzed using the Student's t-test, and multiple group comparisons were performed using one-way or two-way ANOVA. All experiments were repeated at least three times. Intra- and inter-assay CV were maintained below 10% and 15%, respectively. A *P*-value of <0.05 (∗) was considered statistically significant, and <0.01 (∗∗) highly significant.

## 3 Results

### 3.1 RAB5 expression in ovarian GCs is essential for cell proliferation

**Fig. 1:**
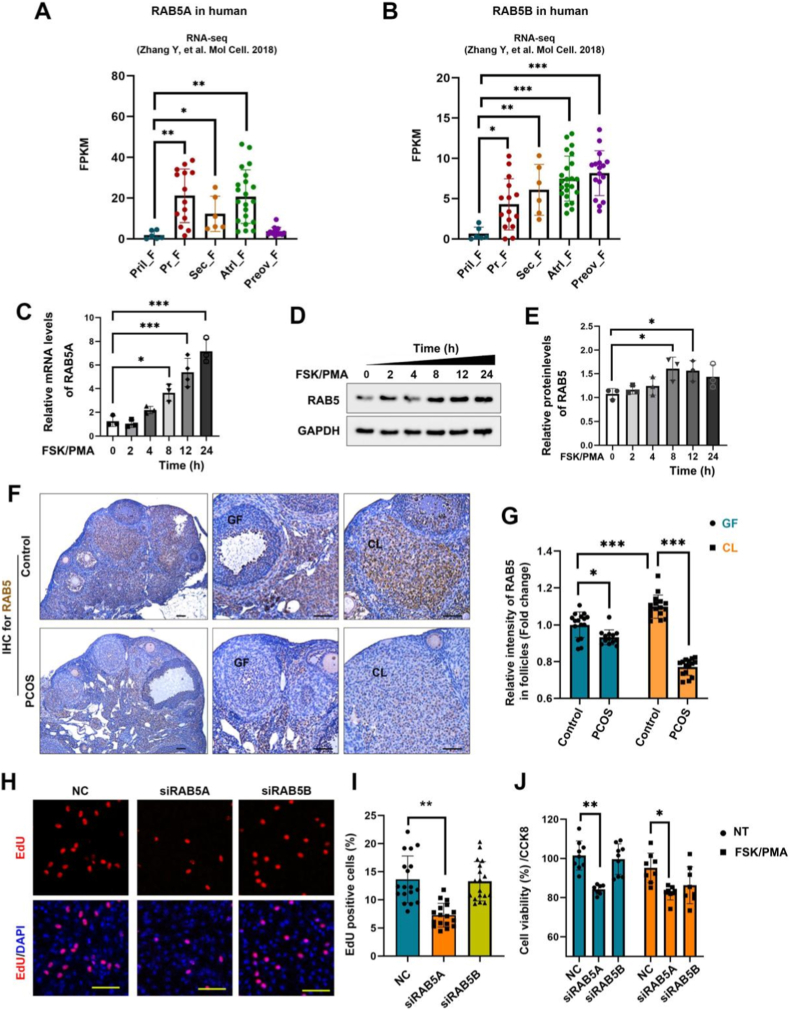
RAB5 expression in ovarian granulosa cells and its regulation on cell proliferation. **A** RNA-seq analysis of RAB5A expression across different human follicular stages. **B** RNA-seq analysis of RAB5B expression across different human follicular stages. **C**Relative mRNA levels of RAB5A following the treatment of FSK/PMA for different time within 0-24 h. **D** Effect of FSK/PMA on RAB5 Protein expression after treatment for different time within 0-24 h. **E** Quantitative analysis of RAB5 expression in WB assay of D. **F** Representative immunohistochemical staining images of RAB5 in ovarian tissues from normal and PCOS mice. Scale bars, 100 μm. **G** Quantitative analysis of RAB5 expression intensity in ovarian tissues from normal and PCOS mice model. **H** Representative EdU staining images of cell proliferation in KGN cells transfected with NC, siRAB5A, or siRAB5B. Scale bars, 100 μm. **I** Statistical analysis of EdU-positive cell proportion in KGN Cells transfected with NC, siRAB5A, or siRAB5B. **J** Cell viability of KGN Cells transfected with NC, siRAB5A, or siRAB5B after NT or FSK/PMA treatment for 24 h. Data are presented as mean ± SD. ∗P < 0.05, ∗∗P < 0.01, ∗∗∗P < 0.001.

To systematically elucidate the roles of RAB5A and RAB5B in ovarian GCs, we first analyzed their expression patterns during human follicular development using previously reported RNA-seq data [23]. RAB5A mRNA levels remained low in GCs from primordial follicles (Pril-F); however, they increased significantly in primary, secondary, and antral follicles (Atrl-F), before declining to baseline in preovulatory follicles (Preov-F) (Fig. 1A). Conversely, RAB5B mRNA expression increased gradually with follicular development, with the lowest levels observed in Pril-F and peaking at Preov-F (Fig. 1B). A key distinction emerged in later stages: RAB5A expression declined in Preov-F while RAB5B reached its peak, although both were highly expressed in Atrl-F. This differential expression suggests functional divergence between the two isoforms and informs subsequent investigations.

Given that RAB5A is implicated in obese PCOS, we next examined its expression during GCs luteinization. KGN cells were treated with the combined treatment of FSK/PMA to induce luteinization, and RAB5A mRNA levels were measured by RT-qPCR. The result indicated significantly increased levels of RAB5A at FSK/PMA treatment for 8, 12, and 24 h (Fig. 1C), indicating its potential involvement in follicular ovulation or luteinization. Furthermore, treatment of cells with FSK/PMA for 0–24 h led to a gradual time-dependent increase in RAB5 protein levels (Fig. 1D and E), suggesting that RAB5 protein expression is upregulated during GC luteinization. To validate RAB5 expression *in vivo*, IHC was performed on ovarian sections from a PCOS mouse model. The PCOS mouse model was constructed through DHEA injections combined with a high-fat diet according to the time graph (Fig. S1A). Successful model establishment was validated by the increased body weight (Fig. S1B), increased ovary weight (Fig. S1C), an abnormal estrus cycle characterized by a persistent estrus period (Fig. S1D), high androgen levels of serum testosterone (T) (Fig. S1E), abnormal glucose metabolism from 0 to 120 min (Fig. S1F), and the polycystic ovarian morphology (Fig. S1G). Compared with the wild-type controls, RAB5 protein expression was significantly reduced in both growing follicles (GF) and corpus luteum (CL) in PCOS ovaries (Fig. 1F). Quantitative analysis further revealed that in normal ovaries, RAB5 expression was significantly higher in CL than in GF (Fig. 1F and G). These findings suggest that RAB5 expression in ovarian tissues exhibits structural specificity and pathological state dependence, and its downregulation may contribute to PCOS development.

Given the distinct expression patterns of RAB5A and RAB5B during folliculogenesis, we next investigated their functional roles in the proliferation and viability of human GCs. KGN cells were modified to knock down RAB5A or RAB5B, and cell proliferation was assessed using an EdU assay. Knockdown of RAB5A, excluding RAB5B, significantly reduced the proportion of EdU-positive cells (Fig. 1H). Quantitative analysis confirmed a marked decrease in the proportion of EdU-positive cells in RAB5A-depleted cells, with no significant change observed upon RAB5B knockdown (Fig. 1I). These results clearly demonstrate that RAB5A, rather than RAB5B, is essential for GCs proliferation. We further evaluated the impact of RAB5 isoforms on cell viability using the CCK-8 assay. Knockdown of RAB5A significantly reduced cell viability, regardless of whether there was 24 h of FSK/PMA treatment. In contrast, RAB5B knockdown exhibited no significant effect on cell viability in either non-treated (NT) or FSK/PMA-treated groups (Fig. 1J). Collectively, these findings underscore a critical and unique role for RAB5A in sustaining proliferation and viability of GCs, whereas RAB5B does not appear to play a central role in these processes. In summary, RAB5A, excluding RAB5B, is indispensable for maintaining GC proliferation and viability.

### 3.2 RAB5A deficiency elevates mitochondrial ROS and induces autophagy

**Fig. 2:**
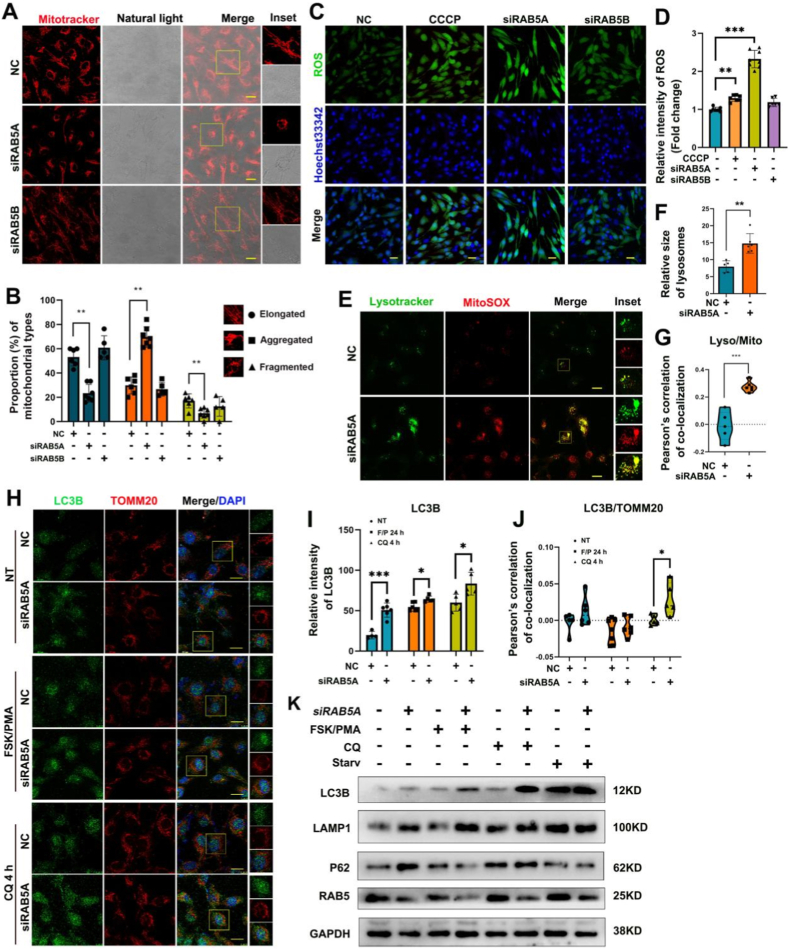
Rab5A deficiency increases mitochondrial ROS, and induces cell autophagy. **A** Effect of RAB5A and RAB5B knockdown on mitochondrial and cell morphology. Scale bars, 20 μm. **B** Statistical analysis of mitochondrial types for data of A. **C** Confocal microscopy observation of reactive oxygen species (ROS) levels after CCCP treatment, or knockdown of RAB5A or RAB5B. Scale bars, 100 μm. **D** Analysis of relative ROS intensity for data of C. **E** Colocalization of lysotracker and MitoSOX staining in RAB5A-knockdown cells to detect mitochondrial ROS levels and lysosome localization. Scale bars, 20 μm. **F** Analysis of lysosome size from data of E. **G** Analysis of lysosome-mitochondria colocalization for data of E. **H** Immunofluorescence staining of LC3B in RAB5A-knockdown cells under different treatments (NT, FSK/PMA, CQ treatments for 4 h). Scale bars, 20 μm. **I** Quantitative analysis of LC3B fluorescence intensity for data of H. **J** Colocalization analysis of LC3B and mitochondrial marker TOMM20 for data of H. **K** Western Blot analysis of autophagy-related protein expression and RAB5 protein levels after RAB5A knockdown under different indicated treatments. Data are presented as mean ± SD. ∗P < 0.05, ∗∗P < 0.01, ∗∗∗P < 0.001.

To further investigate whether RAB5 regulates mitochondrial morphology and function, small interfering RNAs were employed to knockdown RAB5A and RAB5B expression in the human GC line KGN. Alterations in mitochondrial morphology and ROS levels were assessed. The results revealed that RAB5A knockdown markedly altered mitochondrial morphology, significantly increasing the proportion of cells exhibiting mitochondrial aggregation, while reducing the proportions displaying elongated or fragmented mitochondria. Cells with aggregated mitochondria also exhibited a shrunken cellular morphology. In contrast, RAB5B knockdown non-significantly affected mitochondrial morphology or cell morphology (Fig. 2A and B). Since mitochondrial morphology was associated with mitochondrial function, mitochondrial ROS levels were further evaluated using MitoSOX after trypsin digestion. RAB5A knockdown significantly increased the proportion of cells exhibiting elevated mitochondrial ROS, and these cells appeared darker in bright-field imaging. Conversely, no significant changes were observed following RAB5B knockdown (Fig. S2A–B). To further confirm whether RAB5A regulates cellular ROS levels, carbonyl cyanide 3-chlorophenylhydrazone (CCCP) was used as a positive control to induce ROS production. Notably, knocking down RAB5A significantly increased intracellular ROS levels, whereas knocking down RAB5B exhibited a non-significant increase (Fig. 2C and D).

Given that elevated intracellular ROS levels typically enhance lysosomal membrane permeability and impair lysosomal function, we next examined lysosomal size and subcellular localization upon RAB5A knockdown. The results revealed enhanced lysosomal staining, enlarged lysosomes (Fig. 2E and F), and increased colocalization between lysosomes and mitochondria with elevated ROS levels (Fig. 2E–G). Since excess mitochondrial ROS may also trigger apoptosis, we examined whether RAB5A deficiency promotes apoptotic cell death. The results indicated that loss of RAB5A exhibits little effect on apoptosis under basal conditions; however, it significantly increases the proportion of early apoptotic cells following 24 h of FSK/PMA treatment (Fig. S2C–D).

Based on these findings, we hypothesized that RAB5A may interact with mitochondria via a specific pathway and that mitochondrial dysfunction triggers mitophagy to control mitochondrial quality. To explore the mechanism, we analyzed the interaction between autophagosomes and mitochondria using immunofluorescence staining for LC3B and TOMM20 in RAB5A-knockdown cells. RAB5A knockdown significantly increased LC3B fluorescence intensity under basal conditions (NT), following FSK/PMA-induced luteinization, or upon chloroquine (CQ)-mediated inhibition of autophagic flux (Fig. 2H). Quantitative analysis confirmed a marked upregulation of LC3B intensity in RAB5A-depleted cells. However, the extent of increase was attenuated under FSK/PMA or CQ treatment (Fig. 2I). Furthermore, Pearson's correlation analysis of LC3B/TOMM20 colocalization indicated a pronounced alteration in their interaction upon RAB5A knockdown (Fig. 2J). Notably, the colocalization coefficient was substantially enhanced in RAB5A-depleted cells under CQ treatment, suggesting increased targeting of damaged mitochondria by LC3B-positive autophagosomes during mitophagy. Consistently, WB analysis revealed that RAB5A downregulation increased LC3B and P62 protein levels, whereas CQ treatment further increased LC3B levels, while abolished P62 differences (Fig. 2K). Collectively, these results indicate that RAB5A regulates mitophagy by modulating LC3B expression and its mitochondrial recruitment, and that this regulatory function is influenced by luteinization and autophagic flux status.

In conclusion, RAB5A is a critical regulator of mitophagy in ovarian GCs, thereby potentially contributing to mitochondrial quality control and the maintenance of cellular homeostasis.

### 3.3 RAB5 is involved in CCCP-induced mitophagy

**Fig. 3:**
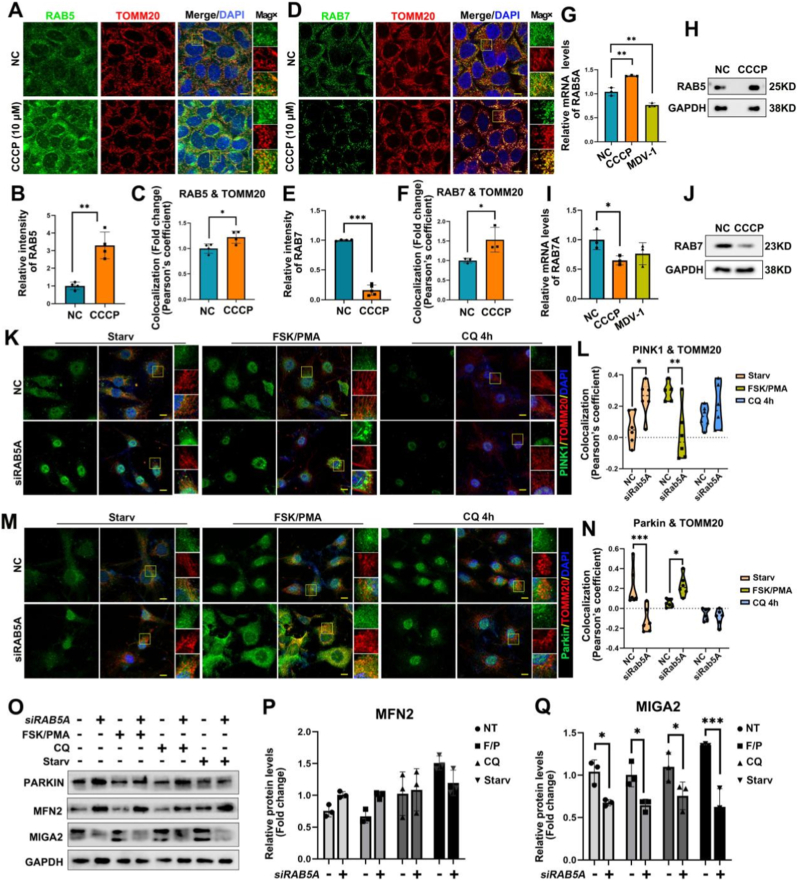
Rab5A regulates mitophagy and is recruited to mitochondria. **A** Representative images of IF staining and colocalization of RAB5 and TOMM20 in cells treated with NC or CCCP for 2 h. Scale bars, 10 μm. **B** Analysis of relative fluorescence intensity of RAB5 for data A. **C** Colocalization analysis of RAB5 and TOMM20 for data of A. **D** Representative images of IF staining and colocalization of RAB7 and TOMM20 in KGN cells treated with CCCP or not. Scale bars, 10 μm. **E** Analysis of relative fluorescence intensity of RAB7 in D. **F** Colocalization analysis of RAB7 and TOMM20 in D. **G** Relative mRNA expression levels of RAB5A in cells treated with NC, CCCP, or MDV-1. **H** WB detection for RAB5 protein expression after the treatment of CCCP or not. **I** Relative mRNA expression of RAB7 in cells treated with NC, CCCP, or MDV-1. **J** WB detection for RBA7 protein expression after the treatment of CCCP or not. **K** Representative confocal microscopy images of immunofluorescence for PINK1 and TOMM20 in RAB5A-deficient cells under different treatments. Scale bars, 10 μm. **L** Quantitative analysis of colocalization coefficient between PINK1 and TOMM20 from data of K. **M**. Representative confocal microscopy images of immunofluorescence for Parkin and TOMM20 in RAB5A-deficient cells under different treatments. Scale bars, 10 μm. **N** Quantitative analysis of colocalization coefficient between Parkin and TOMM20 in K. **O** WB detection of PARKIN, MIGA2 and MFN2 protein levels in RAB5A-deficient cells under different treatments. **P** Quantitative analysis of MFN2 in WB assay of O. **Q** Quantitative analysis of MIGA2 in WB assay of O. Data are presented as mean ± SD. ∗P < 0.05; ∗∗P < 0.01; ∗∗∗P < 0.001.

To investigate endosomal-mitochondrial dynamics under stress, cells were treated with the mitochondrial uncoupler CCCP. CCCP treatment significantly enhanced both the fluorescence intensity and mitochondrial colocalization of RAB5 (Fig. 3A–C), suggesting an upregulation of early endosomes and increased interaction with mitochondria. In contrast, RAB7 exhibited a more complex response. While its overall fluorescence intensity decreased (Fig. 3D and E), its colocalization with mitochondria significantly increased (Fig. 3D–F), implying a reduction in late endosome abundance alongside enhanced mitochondrial association. The mRNA analysis further confirmed that CCCP treatment increased RAB5A while reducing RAB7A mRNA levels. The mitochondrial fission inhibitor Mdivi-1 reduced RAB5A mRNA levels without significantly affecting RAB7A levels (Fig. 3G and H). ​In contrast, immunofluorescence assays indicated that Mdivi-1 increased RAB5 puncta intensity in KGN cells (Fig. S3A–B), indicating a potential role of mitochondrial dynamics on RAB5 expression. Consequently, RAB5 and RAB7 play distinct roles in CCCP-induced mitophagy, suggesting specialized functions for early and late endosomes in mitophagy.

As the canonical mitophagy pathway, the Parkinson Protein 2, E3 Ubiquitin Protein Ligase (PARKIN)-PTEN-induced putative kinase 1 (PINK1) signaling was differentially regulated by RAB5A in a stimulus-dependent manner. Upon starvation, PINK1 demonstrated an increased expression in the nucleus, whereas FSK/PMA treatment promoted its export from the nucleus in RAB5A knockdown cells. (Fig. 3K). Colocalization analysis with Translocase of outer mitochondrial membrane protein 20 (TOMM20) revealed that RAB5A knockdown promoted PINK1 mitochondrial recruitment during starvation (Fig. 3K and L); however, it reduced mitochondrial PARKIN localization (Fig. 3M and N). In contrast, FSK/PMA stimulation triggered the opposite regulatory pattern, with reduced mitochondrial PINK1 localization and increased mitochondrial PARKIN localization (Fig. 3L–N). Notably, overall PARKIN protein expression was non-significantly altered in RAB5A - deleted cells (Fig. 3O–S3C). However, inhibition of autophagy with CQ abolished these differences (Fig. 3K–N). These findings suggest that although the absence of RAB5A can induce PINK1 accumulation on mitochondria, it may ineffectively maintain the stable anchoring or recruitment of PARKIN. However, FSK/PMA-mediated signaling may promote PARKIN recruitment and reduce dependence on PINK1 in the absence of RAB5A. Collectively, these findings indicate that RAB5A is involved in PARKIN-PINK1-mediated mitophagy in KGN cells.

To investigate how RAB5A interacts with mitochondria, the mitochondrial dynamic proteins MFN2 and MIGA2 were examined. WB analysis revealed that RAB5A knockdown increased MFN2 levels—a key regulator of outer mitochondrial membrane (OMM) fusion, regardless of FSK/PMA treatment. However, the lack of RAB5A significantly reduced the expression of MIGA2—another protein involved in OMM fusion and autophagy. This pattern was consistently observed under FSK/PMA treatment, CQ treatment, or starvation conditions (Fig. 3O–Q), suggesting that RAB5A may affect MIGA2 and MFN2-associated functions.

To further assess the spatial relationship between these proteins, we co-transfected HeLa cells with RAB5-mCherry and either MIGA2-GFP or MFN2-GFP. Immunofluorescence imaging and quantitative analysis of Pearson colocalization coefficients significantly increased in spatial overlap between RAB5 and MIGA2. Moreover, the Pearson colocalization coefficient for RAB5 and MIGA2 was significantly higher than that between RAB5 and MFN2 (*P* < 0.05) (Fig. S3D–E).

Given the established role of MIGA2 in regulating mitochondrial membrane contact sites, these findings suggest that RAB5A, beyond modulating the protein levels of MIGA2 and MFN2, preferentially associates with mitochondria-enriched regions via specific spatial interplay with MIGA2. This interaction may facilitate the tethering between mitochondria and endosomal membranes.

### 3.4 RAB5 expression and localization are regulated by MIGA2

**Fig. 4:**
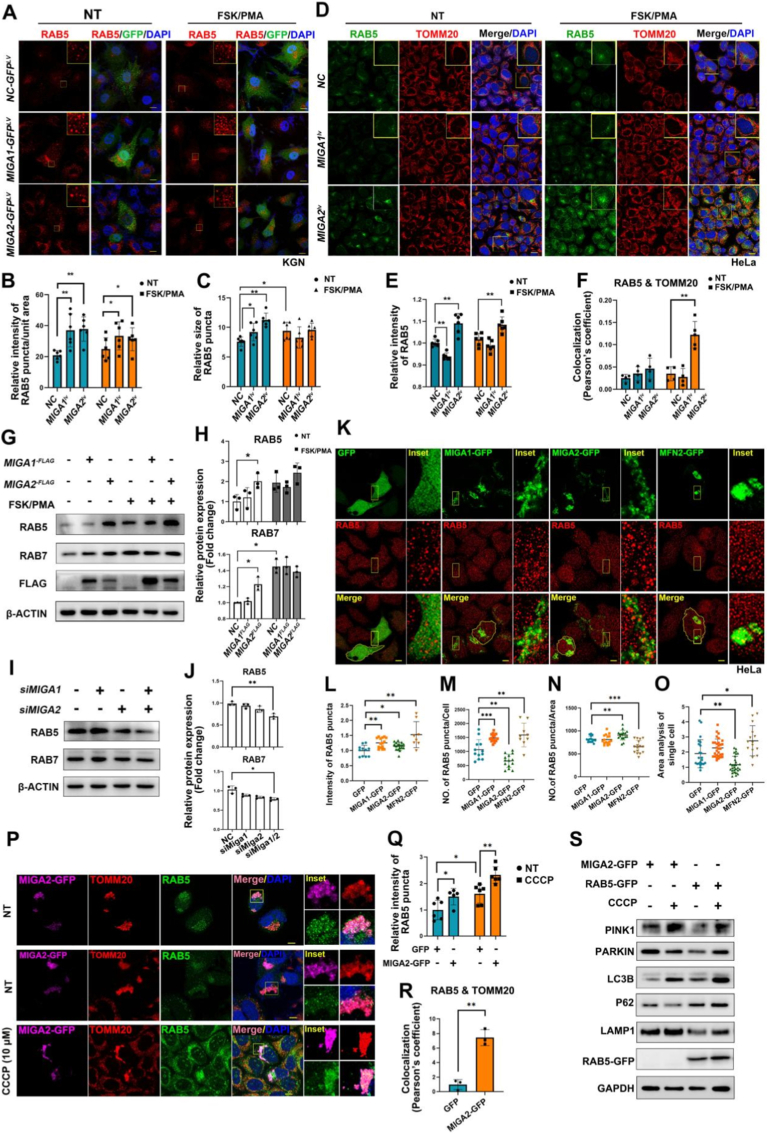
MIGA2 recruits RAB5 to mitochondria and regulates RAB5 expression. **A** Confocal microscopy images of immunofluorescence for RAB5 protein in KGN cells after the expression of MIGA1-GFP or MIGA2-GFP lentiviruses, with or without FSK/PMA stimulation. Scale bars, 10 μm. **B** Quantitative analysis of the relative fluorescence intensity of RAB5 puncta in different groups of KGN cells in A. **C** Quantitative analysis of the relative size of RAB5 puncta in different groups of KGN cells in A. **D** Immunofluorescence detection of the colocalization between RAB5 and the mitochondrial marker protein TOMM20 in HeLa cells after overexpression of MIGA1-GFP and MIGA2-GFP, with or without FSK/PMA stimulation. Scale bars, 10 μm. **E** Quantitative analysis of the relative fluorescence intensity of RAB5 in different groups of HeLa cells in D. **F** Quantitative analysis of the colocalization between RAB5 and TOMM20 in HeLa cells in D using the Pearson's correlation coefficient. **G** WB detection of the protein levels of RAB5, RAB7, and Flag (detection for MIGA1 and MIGA2) in KGN cells with or without FSK/PMA treatments. **H** Quantitative analysis of the relative expression of RAB5 and RAB7 protein in KGN cells shown in G. **I** WB detection of the protein levels of RAB5 and RAB7 in KGN cells after MIGA1,-2 knockdown. **J** Quantitative analysis of the relative expression of RAB5 and RAB7 proteins in KGN cells shown in I. **K** Immunofluorescence detection of the RAB5 protein in HeLa cells after the expression of MIGA1-GFP, MIGA2-GFP, and MFN2-GFP plasmids. Scale bars, 10 μm. **L** Quantitative analysis of RAB5 intensity shown in K. **M** Quantitative analysis of the number of RAB5-stained puncta per GFP-expressing single cell shown in K. **N** Quantitative analysis of the number of RAB5-stained puncta per unit area in GFP-expressing cells shown in K. **O** Quantitative analysis of the area of GFP-expressing single cells shown in K. **P** Immunofluorescence detection of the cellular colocalization between RAB5 and the mitochondrial marker protein TOMM20 in cells after MIGA2-GFP overexpression and CCCP treatment. Scale bars, 10 μm. **Q** Quantitative analysis of the relative fluorescence intensity of RAB5 puncta shown in P. **R** Quantitative analysis of colocalization between RAB5 and TOMM20 using the Pearson's correlation coefficient for data of P. **S** WB analysis of the protein expression of mitophagy-related proteins (PINK1, PARKIN, LC3B, p62, LAMP1) and RAB5-GFP in HeLa cells after overexpression of MIGA2-GFP or RAB5-GFP with CCCP treatment or not. Data are presented as mean ± SD. ∗P < 0.05; ∗∗P < 0.01; ∗∗∗P < 0.001.

To systematically evaluate whether the mitochondrial membrane-associated MIGA proteins regulate RAB5A, stable MIGA1/MIGA2-GFP-overexpressing cells were generated by transfecting the lentiviruses into KGN or HeLa cells. Immunofluorescence staining revealed that, regardless of FSK/PMA stimulation, cells overexpressing MIGA1-GFP or MIGA2-GFP displayed RAB5 aggregation on clustered mitochondria (Fig. 4A). Quantitative analysis of the relative fluorescence intensity of RAB5 puncta further confirmed significantly increased RAB5 protein levels in KGN cells (Fig. 4B), suggesting that MIGA-1 and -2 proteins promote RAB5 expression. In contrast, analysis of the relative size of RAB5 puncta demonstrated a regulatory role of MIGA1 or MIGA2 in RAB5 puncta morphology. Under NT conditions, overexpression of either MIGA1-GFP or MIGA2-GFP significantly enlarged RAB5 puncta; however, this enlargement effect became insignificant following FSK/PMA treatment (Fig. 4C). These findings imply that MIGA1, -2, or FSK/PMA-induced cellular luteinization can influence the morphology of RAB5-associated early endosomes. Similar studies in HeLa cells visualized the expression and distribution of RAB5 relative to the mitochondrial marker TOMM20 by immunofluorescence (Fig. 4D). Quantification of the relative fluorescence intensity of RAB5 further validated the effect of MIGA2 overexpression on RAB5 expression (Fig. 4E). Pearson's coefficient of RAB5 and TOMM20 protein signals indicated a significant RAB5-mitochondria colocalization in MIGA2-overexpressed cells following FSK/PMA treatment (Fig. 4F). Furthermore, MIGA2 overexpression significantly upregulated RAB5 and RAB7 protein levels, while MIGA1 overexpression indicated no significant effect (Fig. 4G and H). These findings indicated that MIGA2 exerts a more prominent positive regulatory effect on RAB family proteins. Furthermore, when MIGA1 and MIGA2 were double-knockdown, the relative protein levels of RAB5 and RAB7 decreased significantly (Fig. 4I and J), underscoring the importance of MIGA proteins, particularly MIGA2, in maintaining RAB protein expression. These findings suggest that RAB5-positive early endosomes may participate in MIGA2-mediated mitochondrial fusion or inter-organelle interactions. This process appears to be influenced by FSK/PMA-induced signaling, which modulates RAB5 expression and the size of its puncta.

To explore the relationship between RAB5 and mitochondrial fusion, we overexpressed MIGA1-GFP, MIGA2-GFP, and MFN2-GFP in HeLa cells, and the subcellular localization of RAB5 was examined upon mitochondrial aggregation. Our results revealed a marked increase in RAB5 protein expression in cells overexpressing MIGA1-GFP, MIGA2-GFP, or MFN2-GFP (Fig. 4K and L). Notably, the number of RAB5-positive early endosomes was elevated in MIGA1-GFP- and MFN2-GFP-overexpressing cells, whereas it decreased in cells overexpressing MIGA2-GFP (Fig. 4M). Despite the reduced number of RAB5-positive endosomes in a single MIGA2-GFP-expressing cell, the density of RAB5 puncta was significantly higher per unit area at mitochondrial aggregation sites (Fig. 4N). Statistical evaluation also indicated a correlation between the number of RAB5 puncta and cell area. Compared with controls, MIGA2-GFP-overexpressing cells exhibited a significant reduction in cell area, whereas MFN2-GFP-overexpressing cells demonstrated an increase (Fig. 4O). Collectively, these findings indicate that MIGA2 overexpression reduces overall cell size while promoting the localized accumulation of RAB5-labeled early endosomes at mitochondrial clusters. This redistribution of RAB5 may contribute to mitochondrial homeostasis and provide valuable insights into the functional and regulatory interplay between RAB5 and MIGA2-mediated mitochondrial function.

Further observations revealed that excessive mitochondrial aggregation driven by MIGA2-GFP overexpression also caused the clustering of RAB5-labeled early endosomes within these regions (Fig. 4P). Quantitatively, the relative fluorescence intensity of RAB5 puncta was significantly higher in MIGA2-overexpressing cells than in unexpressed cells. This effect was similar in cells treated by the mitochondrial uncoupler CCCP (Fig. 4P and Q). RAB5 exhibited strong colocalization with mitochondria, which was significantly enhanced in cells with hyperfused mitochondria (Fig. 4R), indicating that MIGA2 regulates RAB5 distribution at the subcellular level. To determine whether MIGA2 and RAB5A physically interact, a Co-IP assay was performed. However, no specific band corresponding to RAB5A-GFP was detected in the MIGA2-HA immunoprecipitate. In contrast, a positive interaction signal was observed between MIGA2-HA and MIGA2-GFP, which served as an internal control. These results suggest that a direct physical interaction between MIGA2 and RAB5A was undetected under the experimental conditions used (Fig. S4).

WB analysis revealed that CCCP treatment in cells overexpressing MIGA2-GFP or RAB5-GFP activated key mitophagy markers: PINK1 was upregulated, PARKIN was stabilized, LC3B-II levels increased, p62 was degraded, and LAMP1 expression changed accordingly, confirming the activation of autophagic flux (Fig. 4S).

Collectively, our findings demonstrate that MIGA2 might synergistically regulate mitophagy by promoting its colocalization with RAB5, a mechanism that remains active under mitochondrial stress. Besides its functions in mitochondrial fusion and autophagy, MIGA2 contributes to lipidogenesis and also the formation of the endoplasmic reticulum (ER)-mitochondria contact sites (ERMCS) structures. Whether RAB5 participates in lipid metabolism, however, remains to be investigated.

### 3.5 RAB5A regulates lipid metabolism and steroidogenesis in GCs

To determine whether RAB5A regulates lipid metabolism in GCs, we knocked down RAB5A in KGN cells and performed metabolomic analysis. Blank samples were included throughout the experiment, and no distinct peaks for the internal standards were detected in any of the samples, indicating that the levels of residues were low and that cross-contamination between samples was within acceptable limits (Fig. S5A). Three QC samples were included in the experiment, and a Pearson's correlation analysis was performed on the QC samples. All correlation coefficients were above 0.98, indicating a strong correlation among the repeated samples and a good experimental reproducibility (Fig. S5B). ECDF images for all CV indicated that more than 85% of QC samples—both lipid compounds (Fig. S5C) and widely-targeted compounds (Fig. S5D)—exhibited CV values below 0.3, indicating that the experimental data are highly stable. Principal component analysis of the samples (including quality control samples) revealed the overall differences in metabolites among the sample groups and the degree of variability within each group. The results indicated a clear trend of metabolomic separation between control and RAB5 - knockdown groups, and the principal component 1 (43.15%) data indicated obvious separation in the metabolomic profiles between the sample groups (Fig. S5E). This was further confirmed by OPLS-DA (Fig. S5F), which maximized the distinction between the two groups, making it more effective for identifying differentially expressed metabolites.

**Fig. 5:**
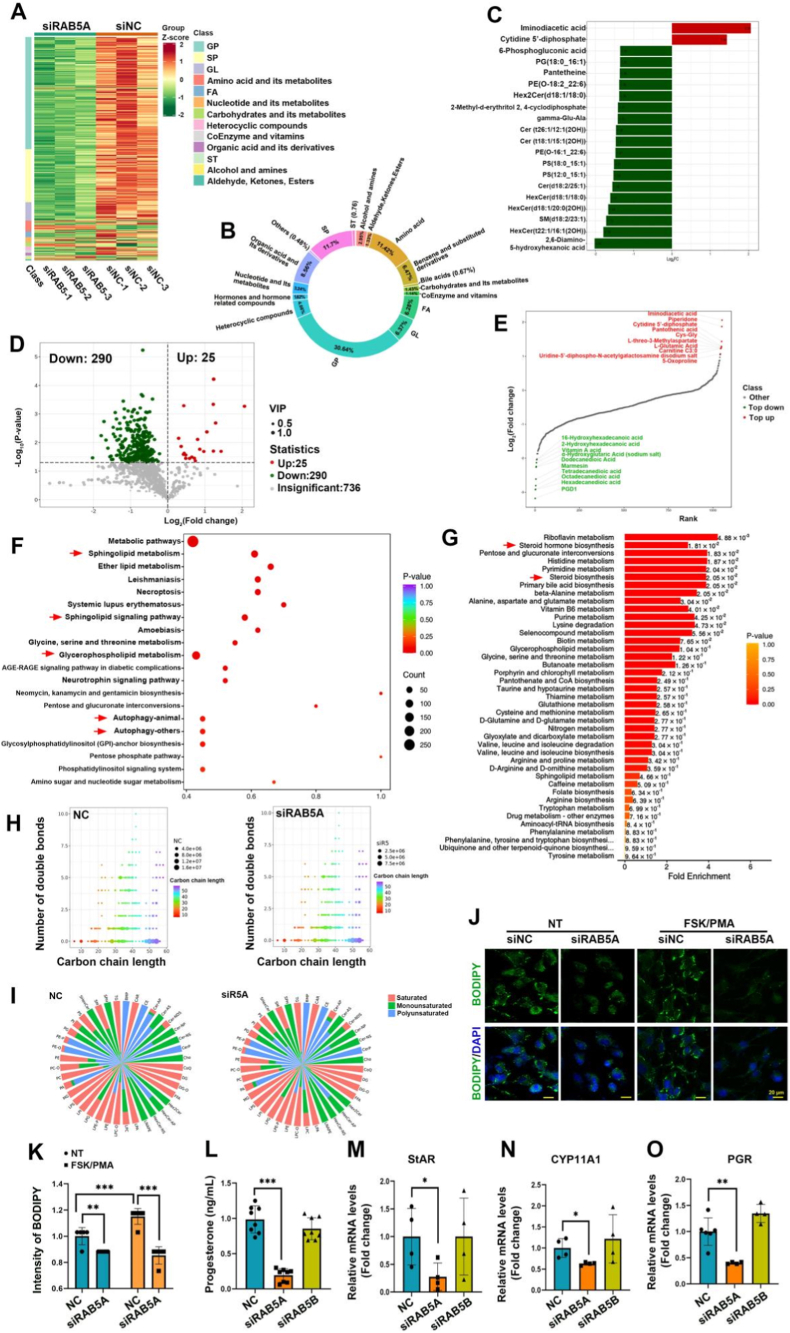
RAB5 regulates lipid metabolism and steroidogenesis in follicular GCs. **A** Hierarchical clustering heatmap of differential metabolites. Colors are color-coded based on normalized values of different relative contents (red indicates high content, and green indicates low content). **B** Proportions of the components of the differential metabolites. **C** Bar chart of fold changes. Red represents upregulated differential metabolites, and green represents downregulated differential metabolites. **D** Volcano plot of differential metabolites. Red represents upregulated differential metabolites, green represents downregulated differential metabolites, and gray represents non-significant differential metabolites. **E** Dynamic distribution plot of metabolite content differences. Dots are arranged in ascending order of fold changes; red represents the top 10 upregulated differential metabolites, and green represents the top 10 downregulated differential metabolites. **F** KEGG enrichment plot of differential metabolites. It shows metabolic pathways with significant enrichment, where the size of dots represents the number of enriched differential metabolites. **G** MSEA enrichment level analysis plot. A higher value indicates a higher enrichment level, and a redder color indicates more significant enrichment. **H** Analysis of lipid chain lengths for total compounds of the NC and siRAB5A KGN cells. **I** Saturation analysis for the total compounds of the NC and siRAB5A KGN cells. **J&K** Representative images of BODIPY 493/503 staining (J) and quantitative analysis (K) of relative intensity of BODIPY in KGN cells transfected with siNC or siRAB5A, followed by treatment with NT or FSK/PMA. Scale bars, 20 μm. **L** ELISA plot of progesterone levels in KGN cells transfected with siRAB5A or siRAB5B. **M-O** Relative mRNA expression analysis of StAR (M), CYP11A1 (N), and PGR (O) in KGN cells transfected with siRAB5A or siRAB5B. Data are presented as mean ± SD. ∗P < 0.05; ∗∗P < 0.01; ∗∗∗P < 0.001.

Hierarchical clustering of the heatmap revealed significant alterations in lipid metabolites. Specifically, the compound levels of 30.64% glycerophospholipids (GP), 11.7% sphingolipids (SP), 6.37% glycerolipids (GL), and 6.28% FA were generally reduced in RAB5A - knockdown cells compared with those in the controls (Fig. 5A and B). Fold-change analysis of the top 20 metabolites revealed two with significant increases, whereas the remaining 18 exhibited notable decreases (Fig. 5C). A volcano plot confirmed 290 significantly decreased and 25 significantly increased metabolites (Fig. 5D), and the top 10 upregulated and downregulated metabolites are displayed in a distribution plot (Fig. 5E). KEGG enrichment analysis indicated significant enrichment in metabolic pathways, including SP metabolism, GP metabolism, and autophagy pathways (Fig. 5F). MSEA further indicated compounds involved in the steroid and steroid hormone biosynthesis (Fig. 5G). Collectively, these data suggest RAB5A regulates steroid hormone synthesis and lipid metabolism in human GCs.

Analysis of lipid chain lengths revealed that the chains of lipids became widely shorter following RAB5A knockdown, especially the phospholipids including the phosphatidic acid (PA), phosphatidylcholine (PC), phosphatidylethanolamine (PE), phosphatidylglycerol (PG), phosphatidylinositol (PI) and phosphatidylserine (PS) (Fig. 5H and S6A-F). And the unsaturated fatty acids (UFAs) levels were widely decreased in the RAB5A-depleted cells, especially the phospholipids including the PA, PC, PE, PG, PI and PS (Fig. 5I and S6G-L). Further analysis revealed no significant changes in the ratios of total polyunsaturated fatty acids (PUFAs) or monounsaturated fatty acids (MUFAs) to saturated fatty acids (SFAs) (Fig. S6M and N). However, significant increases in RAB5A-deficient cells were found in ratio of PCs PUFAs but not in PCs MUFAs to SFAs (Fig. S6O and P). These phospholipids are key for membrane integrity and membrane organelle functions. PCs are important for maintaining membrane fluidity and integrity, PGs stabilize the structure of the mitochondrial inner membrane and support the assembly of electron transport chain complexes. As a structural component of the autophagosome membrane, PE promotes membrane extension and closure. PA serves as a common precursor for the synthesis of other important phospholipids, such as PC, PE, PG, and cardiolipin (CL), which provide the basic framework for the biosynthesis of cell membrane lipids. PS acts as a marker for apoptosis when it is exposed on the outside of the cellular membrane, however, being synthesized at the ERMCS, it might be influenced by the formation of the ERMCS structure. In brief, these phospholipids are major components of cell membranes that are essential for the structural integrity and normal function of mitochondria [24,25].

To functionally validate these findings, we used BODIPY staining to visualize lipid droplet formation. Fluorescence microscopy indicated that the BODIPY signal was significantly weaker in the siRAB5A group than in the controls, regardless of whether there was FSK/PMA stimulation or not (Fig. 5J and K), indicating that RAB5A knockdown reduces lipid droplet levels. Furthermore, Enzyme-Linked Immunosorbent Assay measured significantly reduced progesterone levels in RAB5A-knockdown cells than in the negative control (NC) cells (Fig. 5L). Consistent with this, qRT-PCR analysis demonstrated that RAB5A knockdown significantly reduced the mRNA levels of key steroidogenic genes, including the steroidogenic acute regulatory protein (StAR), cholesterol side-chain cleavage enzyme 11A (CYP11A1), and the progesterone receptor (PGR) (Fig. 5M − O), confirming the regulatory role of RAB5A in the steroid hormone synthesis at the genetic level.

In conclusion, our results demonstrate that RAB5A is a key regulator of steroid hormone synthesis and lipid metabolism in human GCs. We propose a model in which RAB5A maintains membrane formation and transport between endosomes and mitochondria by regulating the synthesis of phospholipids essential for membrane biogenesis and flow. This model posits that RAB5A is essential for producing key lipid metabolites (GP and SP), regulates autophagy to supply lipid substrates for steroidogenesis, and simultaneously upregulates the expression of steroidogenic genes, such as StAR and CYP11A1, to promote hormone synthesis.

### 3.6 RAB5A or MIGA2 regulates AMPK activity in ovarian GCs

**Fig. 6:**
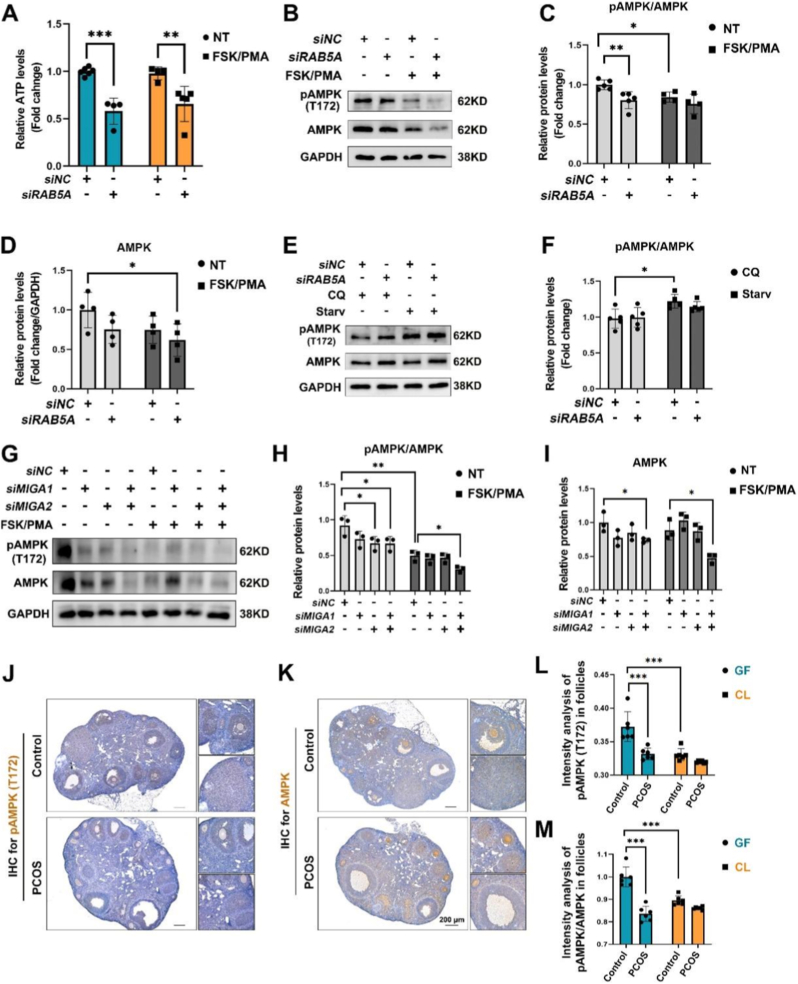
Rab5 and MIGA2 regulates AMPK activity in follicular GCs. **A** Diagram of changes in cellular ATP levels under NC or FSK/PMA treatment after RAB5A knockdown. **B** Western blot detection of pAMPK and AMPK protein expression in KGN cells under RAB5A knockdown and FSK/PMA treatment. **C** Quantitative analysis of the pAMPK/AMPK ratio for data of B. **D** Quantitative analysis of total AMPK protein expression for data of B. **E** Western blot detection of pAMPK and AMPK protein expression in KGN cells under RAB5A knockdown and CQ/starvation treatment. **F** Quantitative analysis of the pAMPK/AMPK ratio for data of E. **G** Western blot detection of pAMPK and AMPK protein expression in KGN cells under MIGA1/MIGA2 knockdown and FSK/PMA treatment. **H** Quantitative analysis of the pAMPK/AMPK ratio for data of G. **I** Quantitative analysis of total AMPK protein expression for data of G. **J**&**K** IHC assay for detection of pAMPK(Thr172) (J); and AMPK (K) on ovarian sections from PCOS and control mice; Scale bars, 200 μm. **L** Quantitative analysis of pAMPK(Thr172) protein levels in growing follicles (GF) and corpus luteum (CL) for data of J. **M** Quantitative analysis of the fold change for the ratio of pAMPK(Thr172) to total AMPK protein levels in GF and CL for data of J and K. Data are presented as mean ± SD. ∗P < 0.05; ∗∗P < 0.01; ∗∗∗P < 0.001.

To determine how RAB5A regulates GC proliferation and lipid metabolism, we assessed cellular energy status and the levels of AMPK signaling molecules in RAB5A-depleted GCs. RAB5A depletion significantly reduced cellular ATP levels, both with and without FSK/PMA stimulation (Fig. 6A), indicating its essential role in ATP production. Under basal conditions, RAB5A knockdown decreased both phosphorylated AMPK (phosphorylation at Thr172) and total AMPK protein, leading to a significantly lower pAMPK/AMPK ratio (Fig. 6B–D). This demonstrates that RAB5A is required to maintain basal AMPK expression and AMPK activation. Following FSK/PMA stimulation, the control (NC) cells exhibited reduced pAMPK and a lower pAMPK/AMPK ratio. In RAB5A-depleted cells, FSK/PMA treatment further reduced total AMPK. Although pAMPK levels decreased slightly, they displayed non-significant differences compared with the NC cells (Fig. 6C and D). Moreover, compared with its own untreated subgroup, FSK/PMA stimulation failed to effectively restore the AMPK activation defect in the siRAB5A group. This suggests that FSK/PMA stimulation amplifies RAB5A's regulatory effect on the AMPK pathway and that RAB5A is an indispensable regulator of FSK/PMA-mediated AMPK activation. However, under nutritional stress (starvation with or without CQ), RAB5A knockdown impaired stress-induced AMPK activation, as the pAMPK/AMPK ratio failed to recover to control levels (Fig. 6E and F). This indicates that RAB5A regulates protein expression and activation efficiency of AMPK, thereby controlling ATP production under basal, FSK/PMA-stimulated, and nutritional stress conditions. RAB5A is a key regulator of cellular energy homeostasis. Accordingly, RAB5A is a key regulator maintaining cellular energy homeostasis.

We next investigated the role of MIGA proteins in AMPK signaling in KGN cells. Under basal conditions, knockdown of MIGA2 or both MIGA1 and -2 reduced the pAMPK/AMPK ratio; FSK/PMA stimulation reduced AMPK phosphorylation in control cells. However, this effect was significantly amplified in MIGA1-and MIGA2-deficient cells, which exhibited a much greater decrease in the pAMPK/AMPK ratio compared to controls (Fig. 6G and H). The total levels of AMPK were also decreased in MIGA1-and MIGA2-deficient cells with or without FSK/PMA treatment (Fig. 6G–I). Based on this, FSK/PMA treatment generally reduces cellular AMPK activity, and the depletion of MIGA1 or MIGA2 further amplifies this inhibitory effect. This indicates that MIGA2 can positively regulate AMPK activity and is critical for maintaining basal AMPK activity under FSK/PMA treatment.

Since RAB5 and MIGA2 expression in GCs are both associated with PCOS, we further detected AMPK and pAMPK (T172) levels *in vivo*. IHC on PCOS mouse models and control mouse ovaries indicated that pAMPK (T172) levels were reduced in PCOS GF; however, no significant changes occurred in CLs (Fig. 6J–L). Furthermore, AMPK activity, calculated as the ratio of pAMPK (T172) to total AMPK protein levels, was significantly decreased in PCOS growing follicles, whereas no significant changes were observed in the corpus luteum (Fig. 6K and M). Collectively, the regulation of AMPK by RAB5A and MIGA2 suggests they may function within interconnected pathways to jointly control GC energy metabolism through the AMPK pathway.

### 3.7 AICAR restores defects caused by RAB5A deficiency

**Fig. 7:**
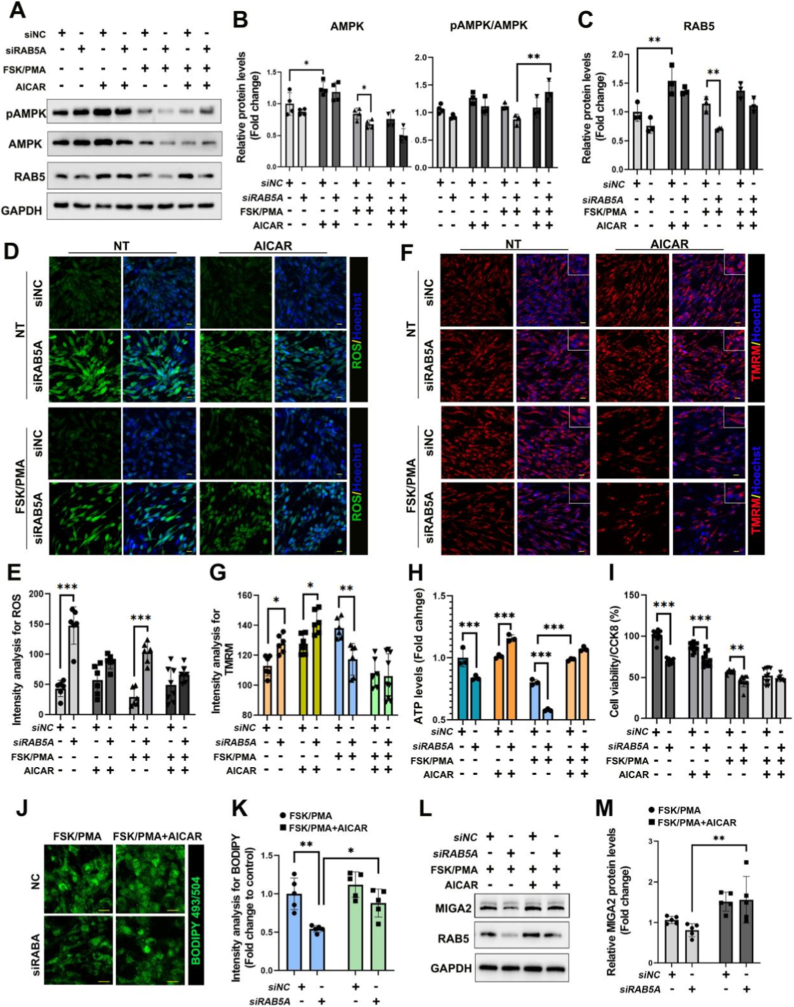
AICAR restores cellular dysfunctions in RAB5-deficient GCs. **A** WB detection of pAMPK, AMPK and RAB5 protein levels in KGN cells under the indicated treatments of FSK/PMA (10 μM/20 nM) or AICAR (0.5 mM) for 16 h. **B** Quantitative analysis of the total AMPK protein levels and the pAMPK/AMPK ratios for data of A. **C** Quantitative analysis of the RAB5 protein levels for data of A. **D** Confocol microscope images for ROS detection through the live imaging assay; Scale bars, 20 μm. **E** Quantitative analysis of ROS for data in D. **F** Confocol microscope images for mitochondrial activity detected by TMRM in RAB5A-deficient KGN cells after FSK/PMA or AICAR treatments. Scale bars, 20 μm. **G** Qualitative analysis of mitochondrial activity for data of F. **H**. ATP levels were rescued after the treatment of AICAR in RAB5A-deficient cells with FSK/PMA treatment or not. **I** CCK-8 assay for detecting the effect of AICAR on cell viability in RAB5A-deficient KGN cells **J** Lipid accumulation detected by BODIPY (493/504) in RAB5A-deficient KGN cells after AICAR treatment. Scale bars, 20 μm. **K** Qualitative analysis of lipid staining intensity for data in J. **L** WB images for MIGA2 and RAB5 protein levels under the indicated treatments of FSK/PMA (10 μM/20 nM) or AICAR (0.5 mM). **M** Quantitative analysis of the MIGA2 for WB data of L. Data are presented as mean ± SD. ∗P < 0.05; ∗∗P < 0.01; ∗∗∗P < 0.001.

To determine whether AMPK could rescue the RAB5A loss-of-function phenotype, AICAR, a compound that can activate AMPK, was applied in the subsequent assays. The results revealed that AICAR effectively increased total AMPK protein levels; however, this effect was abolished under FSK/PMA treatment. Notably, the pAMPK/AMPK ratio remained elevated even in the presence of FSK/PMA (Fig. 7A and B). Similarly, AICAR could increase RAB5 protein levels; however, it failed to do so under FSK/PMA treatment (Fig. 7A–C). However, AICAR reduced the differences in AMPK or RAB5 protein levels caused by RAB5 loss (Fig. 7A–C). Surprisingly, AICAR decreased the high ROS levels induced by lack of RAB5, regardless of whether FSK/PMA treatment was present (Fig. 7D and E). The rescue effects of AICAR on mitochondrial activity were assessed by tetramethylrhodamine methyl ester (TMRM) staining. Mitochondrial activity was significantly increased when RAB5A was knocked down and further elevated under AICAR treatment for 16 h. However, FSK/PMA treatment decreased mitochondrial activity in RAB5A-knockdown cells, and subsequent addition of AICAR triggered a severe suppression of mitochondrial activity, showing no differences between RAB5A-knockdown cells and control cells (Fig. 7F and G). These results indicate that RAB5A is indispensable for maintaining mitochondrial homeostasis during GC luteinization.

To distinguish whether RAB5A directly regulates AMPK or whether the decline in AMPK activity is merely a secondary consequence of cellular energy collapse, we utilized the AMPK agonist AICAR in RAB5A-deficient cells. The results revealed that AICAR-induced AMPK activation significantly increased ATP levels (Fig. 7H). This key finding indicates that, in the context of RAB5A deficiency, the AMPK activation pathway remains intact and functionally competent, and can positively regulate energy metabolism. Accordingly, the reduction in AMPK activity caused by RAB5A depletion is unlikely to be solely a passive outcome of energy depletion. Instead, it favors the hypothesis that RAB5A acts upstream of AMPK, exerting a direct or indirect positive regulatory role to maintain normal cellular energy homeostasis.

Importantly, AICAR effectively rescued the impaired cell viability caused by RAB5A knockdown, restoring it to near-normal levels, especially in cells with a combined treatment of FSK/PMA or AICAR (Fig. 7I). Furthermore, AICAR restored the levels of neutral lipid droplets that were reduced after FSK/PMA-induced luteinization (Fig. 7J and K). In summary, the ability of the AMPK activator AICAR to ameliorate the phenotypes of RAB5A deficiency—specifically, reduced cell viability and lipid droplet—indicates that AMPK acts as a significant downstream pathway in the RAB5A signaling network. To dissect the regulatory hierarchy linking RAB5A, AMPK, and MIGA2, we stimulated AMPK activity with AICAR in RAB5A-deficient KGN cells. We found that pharmacological AMPK activation rescued MIGA2 protein expression despite FSK/PMA challenge (Fig. 7L and M), demonstrating that AMPK operates upstream of MIGA2 in a RAB5A-independent manner. Furthermore, AMPK signaling can functionally substitute for RAB5A loss to restore both MIGA2 expression and mitochondrial homeostasis. These data establish a RAB5A-AMPK-MIGA2 signaling axis governing granulosa cell mitochondrial function.

## 4 Discussion and conclusions

Previous studies have established that GCs provide energy for lipid metabolism via mitochondrial β-oxidation, and RAB5 can regulate mitochondrial homeostasis through its effectors, such as FERRY [10,26]. GCs from patients with PCOS exhibit decreased RAB5A expression and disrupted lipid metabolism [18,27]; however, the causal relationship and underlying molecular mechanisms linking these abnormalities remain unverified. As a cellular energy sensor, AMPK can mediate the coupling between mitochondrial function and metabolic processes [28]. Here, we report a novel mechanism by which RAB5A regulates mitochondrial functions and ROS levels, ultimately modulating cell proliferation and lipid metabolism via AMPK/MIGA2 signals in GCs. In this study, we identified RAB5A as a key regulator of mitochondrial function and lipid metabolism in GCs. RAB5A modulates mitochondrial morphology, mitochondrial ROS levels, and autophagic activity by regulating AMPK activity, thereby maintaining the homeostasis of lipid metabolites—including GP and SP—as well as the synthesis of steroid hormones such as progesterone. Furthermore, treatment with AICAR, which can activate AMPK, significantly reverses the decreased cell viability and lipid metabolic disorders induced by RAB5A deficiency. This finding bridges the gap in understanding the crosstalk among endosomes, mitochondria, and lipid droplets in GCs via the RAB5-AMPK-MIGA2 signaling pathway. It offers a novel molecular perspective for deciphering the pathological mechanisms of PCOS.

Our study revealed that RAB5A deficiency in GCs led to significantly increased ROS levels, elevated LC3B-II expression, and impaired P62 degradation. Furthermore, loss of RAB5A affected the colocalization of the core mitophagy factors PINK1 and PARKIN with mitochondria. These findings are consistent with previous reports that RAB5 regulates mitochondrial quality by modulating autophagy in a PARKIN-dependent manner [29]. However, whether RAB5A directly regulates AMPK-mediated mitochondrial functional repair by influencing PINK1 phosphorylation, or whether it mediates mitochondrial recruitment of PARKIN, requires further investigation.

Previous studies have reported that AMPK can promote lipid droplet-mitochondria crosstalk via Rab8a, accelerating fatty acid β-oxidation [12]. Additionally, the OMM protein MIGA2 regulates mitochondrial fusion, ERMCS, and autophagy [21,30,31], and contributes to cell proliferation, lipid droplet transfer, and synthesis [20,32,33]. In our study, RAB5A knockdown in GCs led to decreased cell proliferation, significantly reduced levels of GL, GP, and SP, and markedly reduced progesterone levels, along with downregulation of key steroidogenic genes (StAR, CYP11A1, and PGR). These findings confirm the central role of RAB5A in lipid metabolism and steroidogenesis and further suggest that RAB5A may regulate the transport of lipid droplet substrates to mitochondria through the AMPK/MIGA2 signaling axis. Lipid droplets can undergo lysosomal degradation via the endosomal pathway. RAB5, a key regulatory protein for endosomal maturation, is deficient, leading to the accumulation of Rab5-Rab7 double-positive intermediate endosomes that block lipid droplet clearance and trigger triacylglycerol elevation [34]. This is highly consistent with the disrupted lipid metabolism phenotype observed in RAB5A-depleted GCs in our study, suggesting that RAB5A may regulate lipid homeostasis through two distinct pathways: mitochondrial β-oxidation and endosome-mediated lipid droplet degradation. The former maintains mitochondrial function via AMPK to ensure lipid-derived energy supply, while the latter promotes lipid clearance through endosomal trafficking. These two pathways may work together to maintain lipid balance in GCs. This potential mechanism further strengthens the core hub role of RAB5A in organelle crosstalk and provides multi-dimensional exploration directions of vesicular transport-mitochondrial metabolism-lysosomal degradation for subsequent studies.

In this study, although the colocalization of RAB5 with mitochondria was increased in luteinized GCs, experimental evidence demonstrated no direct physical interaction between RAB5A and MIGA2. Although RAB5A deficiency reduces AMPK activity and MIGA2 expression, AMPK activation can bypass RAB5A deficiency to restore MIGA2 expression and mitochondrial function. Conversely, MIGA2 knockout blocks downstream effects and may inhibit AMPK activity through feedback suppression, forming a negative feedback loop that creates a functional dependency. However, the specific mechanism still needs in-depth exploration. For example, whether RAB5A affects the activity of AMPK through direct binding to upstream kinases of AMPK, or promotes the mitochondrial localization of AMPK by regulating MIGA2. Clarifying these links will provide a more complete explanation of the molecular mechanisms underlying RAB5A-AMPK-MIGA2 signaling pathways. In addition, although the phenotype was rescued, the paradox of AMPK being partially “inactivated” under FSK/PMA treatment persists. We propose that contradiction may reflect a differential substrate sensitivity and allosteric regulation rather than AMPK-independent effects. FSK/PMA activate PKA signaling pathway, and the PKA-mediated phosphorylation of AMPKα Ser173 reduces but does not abolish AMPK activity, and residual activity toward specific substrates (e.g., ULK1) may suffice for functional rescue [35]. The phosphorylated metabolite of AICAR—5-Aminoimidazole-4-carboxamide ribonucleotide (ZMP)—binds the γ-CBS sites of the AMPK-γ subunit. This binding activates AMPK kinase activity through conformational changes and potentially overcomes PKA-mediated inhibition through enhanced allosteric protection [36]. This interpretation is consistent with the “threshold" model of kinase signaling, where sub - maximal activity achieves full physiological output [37]. Overall, RAB5A-AMPK-MIGA2 regulatory axis identified in this study indicates that targeting AMPK can restore mitochondrial function independently of RAB5A expression, offering a novel potential therapeutic strategy for treating obese PCOS.

From the perspective of clinical translation, the core findings of this study, centered on the “RAB5A-AMPK-mitochondria" axis, have potential clinical translational value. On the one hand, in terms of potential molecular markers, studies have confirmed a significant correlation between decreased RAB5A expression and reduced AMPK activity in GCs of PCOS. This suggests that RAB5A may serve as a specific molecular marker for evaluating the metabolic function of GCs. RAB5A expression in follicular fluid can be detected and, combined with the phosphorylation status of AMPK in GCs, can assist in evaluating the follicular development potential of patients with PCOS. On the other hand, in terms of potential therapeutic targets, existing studies have confirmed that GCs from patients with PCOS generally exhibit reduced AMPK activity [38], suggesting that AMPK activators are promising candidate intervention agents to improve GC function in patients with PCOS. From a clinical treatment perspective, activating the AMPK signaling pathway may break the vicious cycle of “mitochondrial dysfunction-lipid metabolism disorder-follicular development impairment" triggered by RAB5A deficiency. This provides a potential regulatory node in the pathophysiology of obese PCOS.

In addition, studies have demonstrated that AICAR can activate AMPK and its downstream signaling pathways in a manner similar to metformin and the AMPK activator A769662 [14]. Our study also confirmed that AICAR treatment reduced elevated ROS levels, enhanced mitochondrial activity, and partially restored cell proliferation and lipid metabolism in RAB5A-deficient cells—effects consistent with AMPK-mediated effects [[39], [40], [41], [42]]. Although these findings support a role for AMPK activation in mediating these effects, and the concentration employed (0.5 mM) falls within the commonly used range for AMPK activation, intracellular accumulation of the ZMP at this concentration may exert off-target effects on other AMP-sensitive enzymes. Consequently, definitive establishment of AMPK dependence requires future studies utilizing genetic ablation of AMPK catalytic subunits (AMPKα1/α2 knockdown or knockout) or more specific AMPK activators (PF-06409577 or MK-8722) [43,44]. Besides, metformin is a widely used clinical drug that can stimulate AMPK activation. It is expected to restore the mitochondrial dysfunctions and the disruption of AMPK signaling caused by low expression of RAB5.

Apart from the above, this study has some other limitations. The cells used in this study primarily rely on KGN cells, a tumor-derived GC line that cannot fully mimic primary human GC biology. Further verification is required regarding the *in vivo* function of RAB5A in GC proliferation and lipid metabolism during follicle development and ovarian endocrine function. It is worthwhile to investigate the potential PCOS phenotype by generating RAB5A knockout models in GCs.

In summary, this study clarifies the mechanism by which the RAB5A-AMPK-MIGA2 axis regulates cell proliferation and lipid metabolism by modulating mitochondrial function in GCs. This work systematically demonstrates that endosome-mediated RAB5A functions regulate mitochondrial function and cell autophagy, modulating the AMPK pathway and lipid metabolism in GCs, thereby improving the molecular theoretical system governing GC proliferation and metabolic regulation during follicular development. However, it also provides insights into the pathological mechanisms for PCOS. Abnormalities in the “RAB5A-AMPK-mitochondria" axis may be a key trigger for follicular development disorders, and AICAR may be a potential strategy for treating follicular developmental and ovulatory disorders in PCOS. These findings offer new targets for potential interventions in PCOS accompanied by lipid metabolic abnormalities.

## Ethical approval statement

This study was reviewed and approved by the Animal Care & Welfare Committee of Shandong Provincial Hospital affiliated to Shandong First Medical University (Approval No. 2025-051). All procedures involving animals were conducted in compliance with the ethical standards set forth by the aforementioned committee.

## CRediT authorship contribution statement

**Shao-Hong Liu:** Data curation, Methodology, Writing – original draft. **Ping Yang:** Data curation, Funding acquisition, Methodology, Writing – original draft. **Bing-Hong Zhu:** Data curation, Methodology. **Hong-Yu Li:** Data curation, Methodology. **Shan Wang:** Supervision, Writing – review & editing. **Yong Wang:** Supervision, Writing – review & editing. **Xiao-Man Liu:** Conceptualization, Data curation, Funding acquisition, Methodology, Supervision, Writing – review & editing.

## Declaration of competing interest

The authors declares no conflict of interest.
