# Supplementary material for: RAB5A regulates cell proliferation and lipid metabolism by modulating mitochondrial ROS via AMPK signaling pathway in ovarian granulosa cells
Source: Redox Biol. 2026 Apr 29;93:104183. doi: 10.1016/j.redox.2026.104183 (PMC13144571; doi:10.1016/j.redox.2026.104183)
Supplement: Multimedia component 1 [file mmc1.docx]

**Supplementary files：**

**RAB5 Regulates Cell Proliferation and Lipid Metabolism by Modulating Mitochondrial ROS via AMPK Signaling Pathway in Ovarian Granulosa Cells**

Shao-Hong Liu^1, 2, #^, Ping Yang^3, #^, Bing-Hong Zhu^4^, Hong-Yu Li^3, 5^, Shan Wang^3, 5^, Yong Wang^1, *^, Xiao-Man Liu^2, *^

^1^ Department of Clinical Laboratory Medicine, Shandong Provincial Hospital Affiliated to Shandong First Medical University; Jinan, 250021, China.

^2^ Central Laboratory, Shandong Provincial Hospital Affiliated to Shandong First Medical University, Jinan, 250021, China.

^3^ Department of Obstetrics and Gynecology, Shandong Provincial Hospital Affiliated to Shandong First Medical University, Jinan, 250021, China.

^4^ Department of Obstetrics and Gynecology, The First Affiliated Hospital of Shandong First Medical University& Shandong Provincial Qianfoshan Hospital, Jinan, 250014, China.

^5^ Department of Reproductive Medicine, Shandong Provincial Hospital Affiliated to Shandong First Medical University, Jinan, 250021, China.

^#^ These authors contributed equally.

*Correspondence: Yong Wang: [[sdwangyong@126.com](mailto:sdwangyong@126.com);](mailto:ctong@zju.edu.cn;) Xiao-Man Liu: [liuxiaoman@sdfmu.edu.cn](mailto:liuxiaoman@sdfmu.edu.cn).

**Data included in this file:**

**Tables: S1-S2**

**Figures: S1-S6**

**Table S1. Primers used in quantitative PCR analysis.**

| Gene symbol | Gene ID (NCBI) | Forward primer  (5’-3’) | Reverse primer  (5’-3’) | Primer length |
| --- | --- | --- | --- | --- |
| RAB5A | 5868 | CAACGGGCCAAATACGGGAA | TGGGTTAGAAAAGCAGCCCC | 153 bp |
| RAB7A | 7879 | GTTCCAGTCTCTCGGTGTGG | TTGAATGTGTTGGGGGCAGT | 84 bp |
| StAR | 6770 | CCCCGTGACTTTGTGAGC | CGTAAGTTTGGTCTTAGAGGGA | 180 bp |
| CYP11A1 | 1583 | GATTACCGTGGCATCCTCTAC | CAGCATATCCTGCACCTTCA | 168 bp |
| PGR | 5241 | CAAGTTAGCCAAGAAGAGTTC | ACTTCGTAGCCCTTCCAAAG | 78 bp |
| GAPDH | 2597 | TCTGCTCCTCCTGTTCGACA | AAAAGCAGCCCTGGTGACC | 141 bp |

**Table S2. Antibodies applied in this study.**

| **Antibodies** | **RRID** | **Source company** | **Catalog number** | **Working concentration** |
| --- | --- | --- | --- | --- |
| LAMP1 | AB_2687579 | Cell Signaling Technology Inc. | 9091 | WB: 1:1000 |
| FLAG | AB_10950495 | Cell Signaling Technology | 8146 | WB: 1:1000 |
| MIGA2 (FAM73B) | AB_3086170 | ProteinTech | 29811-1-AP | WB: 1:1000 |
| [P62/SQSTM1](https://rrid.site/data/record/nif-0000-07730-1/AB_10694431/resolver?q=*&l=*&filter%5b%5d=Vendor:ProteinTech&filter%5b%5d=Catalog%20Number:18420-1-AP&i=rrid:ab_10694431-737150) | AB_10694431 | ProteinTech | 18420-1-AP | WB: 1:1000 |
| MFN2 | AB_2266320 | ProteinTech | 12186-1-AP | WB: 1:1000 |
| AMPK | AB_330331 | Cell Signaling | 2532 | WB: 1:1000  IHC: 1:200 |
| pAMPK(Thr172) | AB_331250 | Cell Signaling | 2535 | WB: 1:1000  IHC: 1:200 |
| [Parkin](https://rrid.site/data/record/nif-0000-07730-1/AB_2878005/resolver?q=*&l=*&filter%5b%5d=Vendor:ProteinTech&filter%5b%5d=Catalog%20Number:14060-1-AP&i=rrid:ab_2878005-2813461)/PARK2 | AB_2878005 | ProteinTech | 14060-1-AP | WB: 1:1000 |
| PINK1 | AB_2879244 | ProteinTech | 23274-1-AP | WB: 1:1000 |
| RAB5 | AB_2300649 | Cell Signaling Technology | 3547 | WB: 1:1000  IHC: 1:200 |
| RAB7 | AB_1904103 | Cell Signaling Technology | 9367 | WB: 1:1000 |
| TOMM20 | AB_945896 | Abcam | ab56783 | IF:1:1000 |
| LC3B | AB_2137737 | ProteinTech | 14600-1-AP | WB: 1:1000  IF:1:500 |
| GAPDH | AB_2107436 | ProteinTech | 60004-1-Ig | WB: 1:1000 |
| [Beta-](https://rrid.site/data/record/nif-0000-07730-1/AB_2687938/resolver?q=*&l=*&filter%5b%5d=Vendor:ProteinTech&filter%5b%5d=Catalog%20Number:66009-1-Ig&i=rrid:ab_2687938-2623394)  ACTIN | AB_2687938 | ProteinTech | 66009-1-Ig | WB: 1:1000 |

**
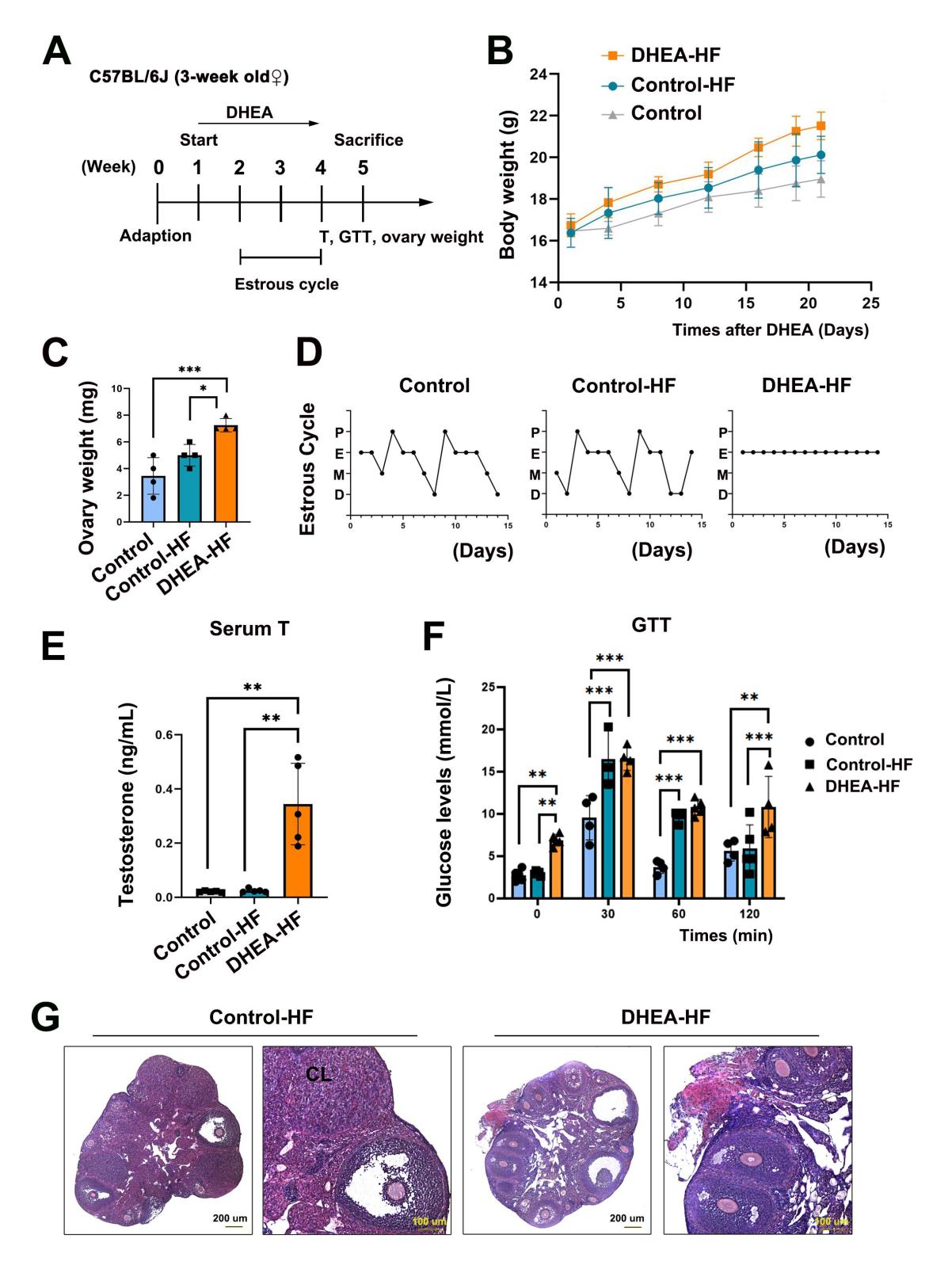
**

**Figure S1. Method and verification data of DHEA-induced PCOS models.**

**A.** Scheme of the method for the construction of DHEA-induced PCOS mouse model. **B.** Changes in body weight during DHEA or sesame oil administration in the DHEA-injection group, mice on a high-fat (HF) diet (DHEA-HF); sesame oil–injection group, mice on a HF diet (Control-HF), and the normal control group (Control) that received no injections and followed a normal diet. **C.** The ovary weight for the three mouse models. **D.** Estrus cycles for the indicated three groups. ‌Proestrus ‌(P),‌ Estrus ‌(E),‌ Metestrus ‌(M), ‌Diestrus (D). **E.** Serum T in the indicated three groups. **F.** Glucose tolerance test **(**GTT) for the indicated three groups. Glucose levels were detected at different times after injection of glucose of 0, 30 min, 60min and 120 min. **G.** Representative images of the ovarian morphology for control-HF and DHEA-HF groups. Data are presented as mean±SD. ***P*<0.01; ****P*<0.001.


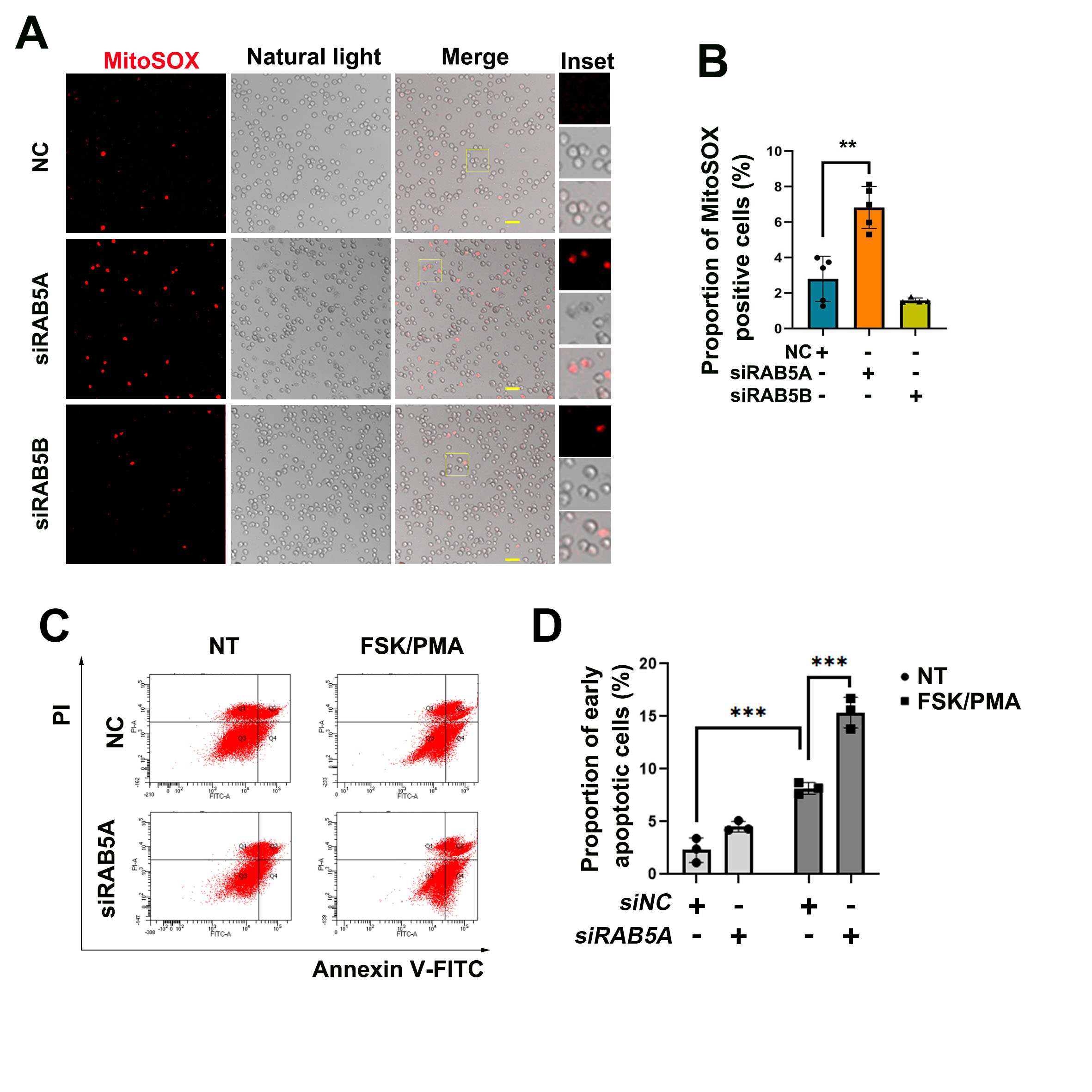


**Figure S2. Loss of RAB5A induces cell apoptosis in luteinized KGN cells.**

**A** Confocal microscopy observation of mitochondrial reactive oxygen species (ROS) levels via MitoSOX staining after RAB5A and RAB5B knockdown. Scale bars, 100 μm. **B** Quantification for the proportion of MitoSOX-positive cells in data of A. **C** Representative flow cytometry images for the indicated treatment in KGN cells. **D** Quantification for the proportion of early apoptotic cells for data A. Data are presented as mean±SD. ***P*<0.01; ****P*<0.001.


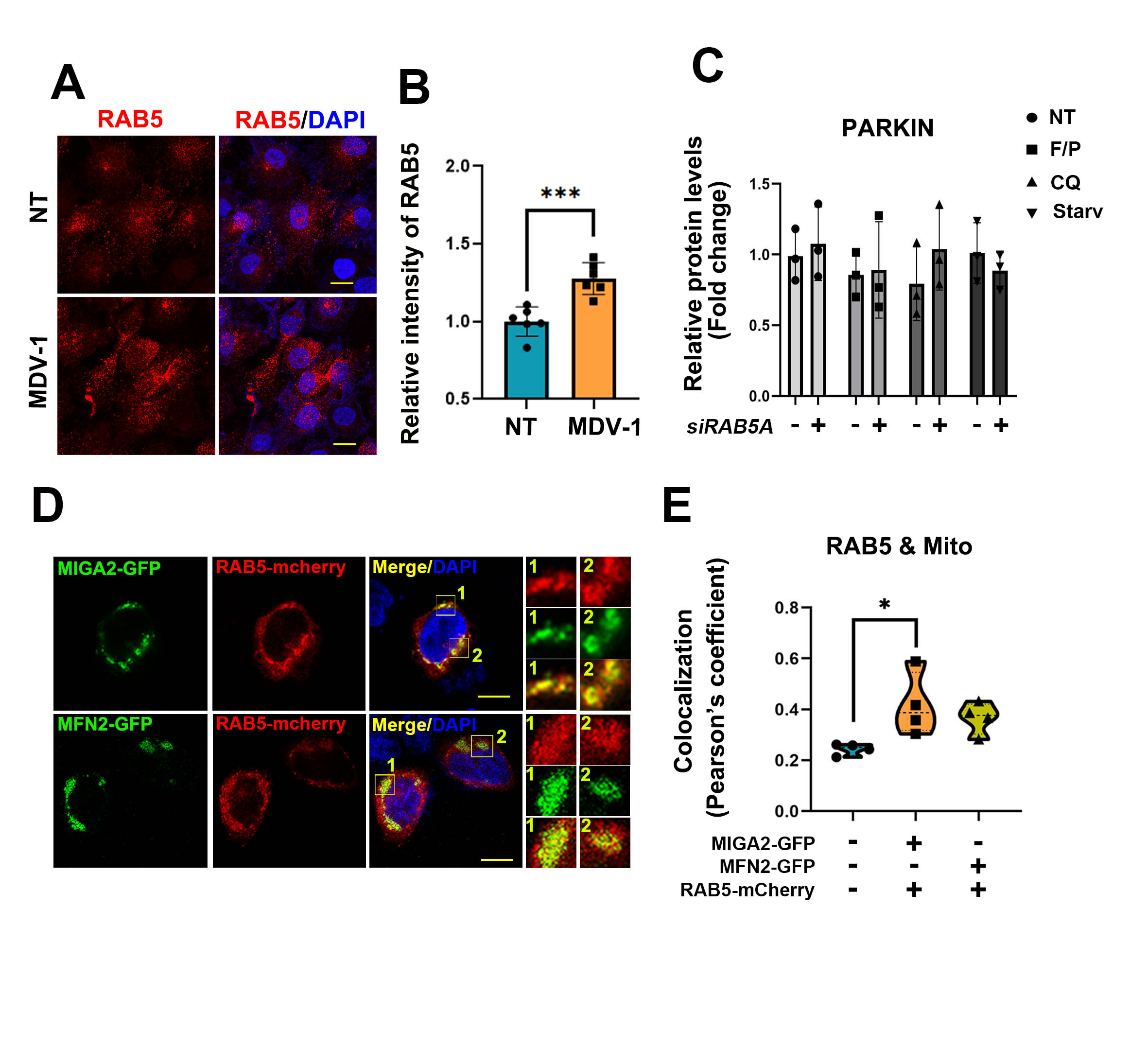


**Figure S3. Rab5 expression and mitochondrial localization were regulated by mitochondrial dynamics.**

**A** The small-molecule compound Mdivi-1 (MDV-1), a mitochondrial fission inhibitor, increased RAB5 protein expression in KGN cells. Scale bars, 20 μm. **B** Quantification analysis for RAB5 expression intensity for data in A. **C** Quantification for the WB analysis for PARKIN in Figure 3O. **D** Confocal microscope images of co-expression of RAB5-mCherry (Red) with MIGA2-GFP or MFN2-GFP. Scale bars, 100 μm. **E.** Quantitative analysis of colocalization coefficient between RAB5 and mitochondrial (Mito) proteins of MIGA2 and MFN2. Data are presented as mean±SD. **P*<0.05; ****P*<0.001.


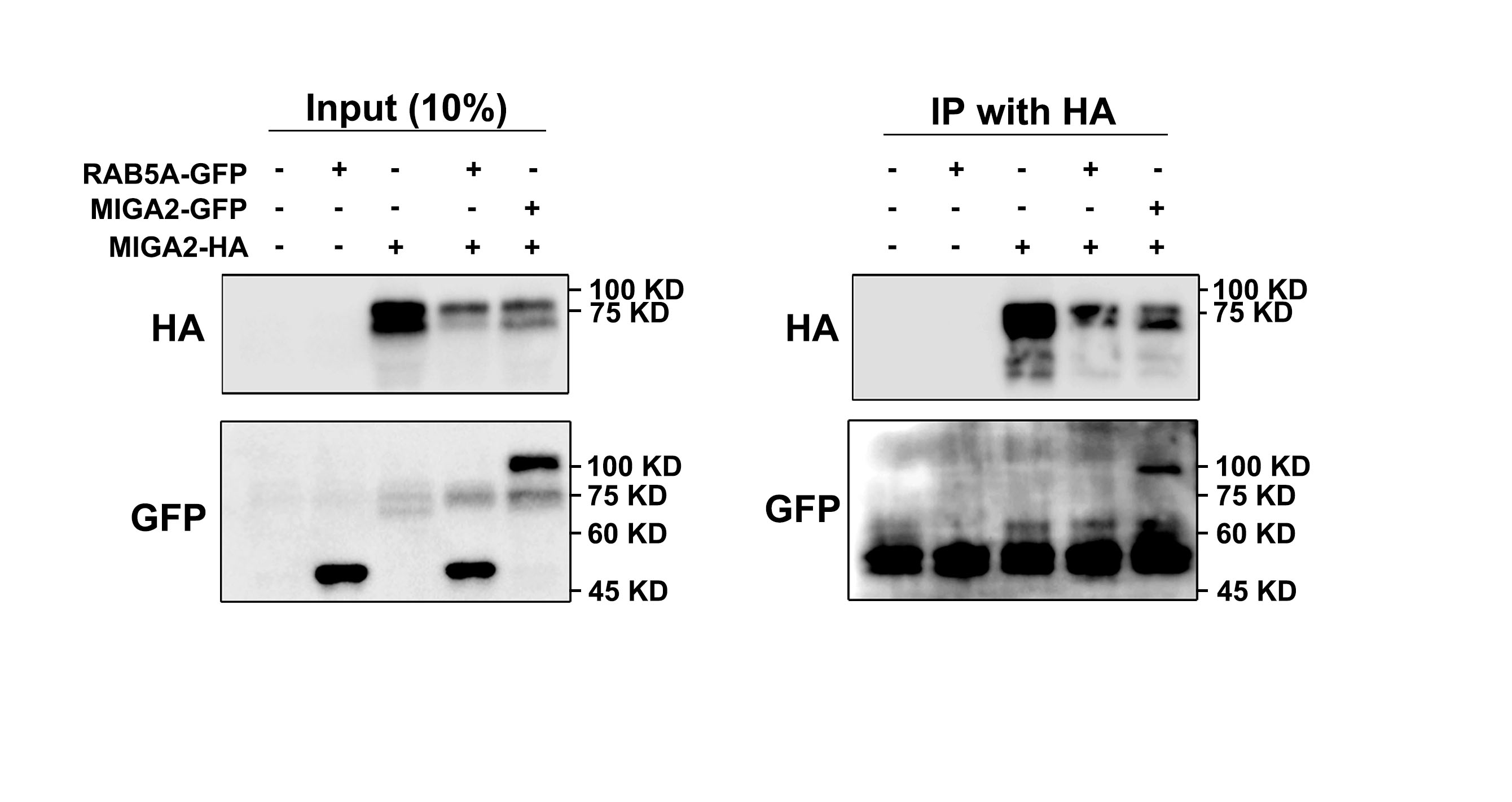


**Figure S4. There is no direct interaction between RAB5 and MIGA2.**

Co-immunoprecipitation (Co-IP) experiments were conducted to detect interactions between the MIGA2 and RAB5A proteins. The results showed that positive bands were detected between MIGA2-HA and MIGA2-GFP, but no positive bands were detected between RAB5-GFP and MIGA2-HA.

**
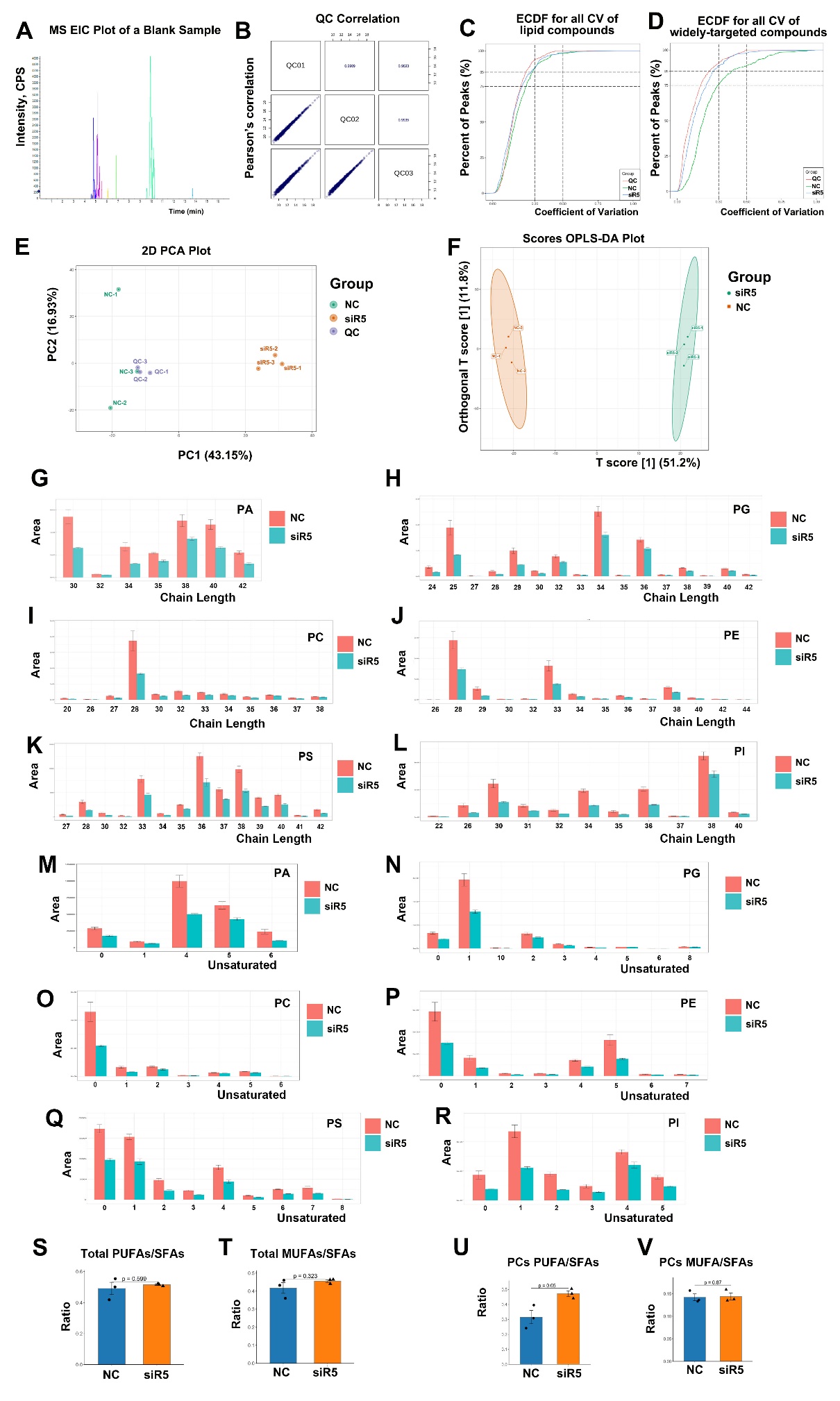
Figure S5. Evaluation of data results of metabolomic analysis.**

**A** Representative LC/MS image for blank samples, and no distinct peaks were detected. **B** Pearson’s correlation analysis for the quality control (QC) samples. **C** ECDF for all CV of lipid compounds in samples of the negative control (NC), siRAB5A (siR5) and the QC. **D** ECDF for all CV of widely-targeted compounds. **E** 2D principal component analysis (PCA) plot of the samples including the NC, siR5 and the QC. **F** OPLS-DA plot for the samples of the NC and siR5.

**
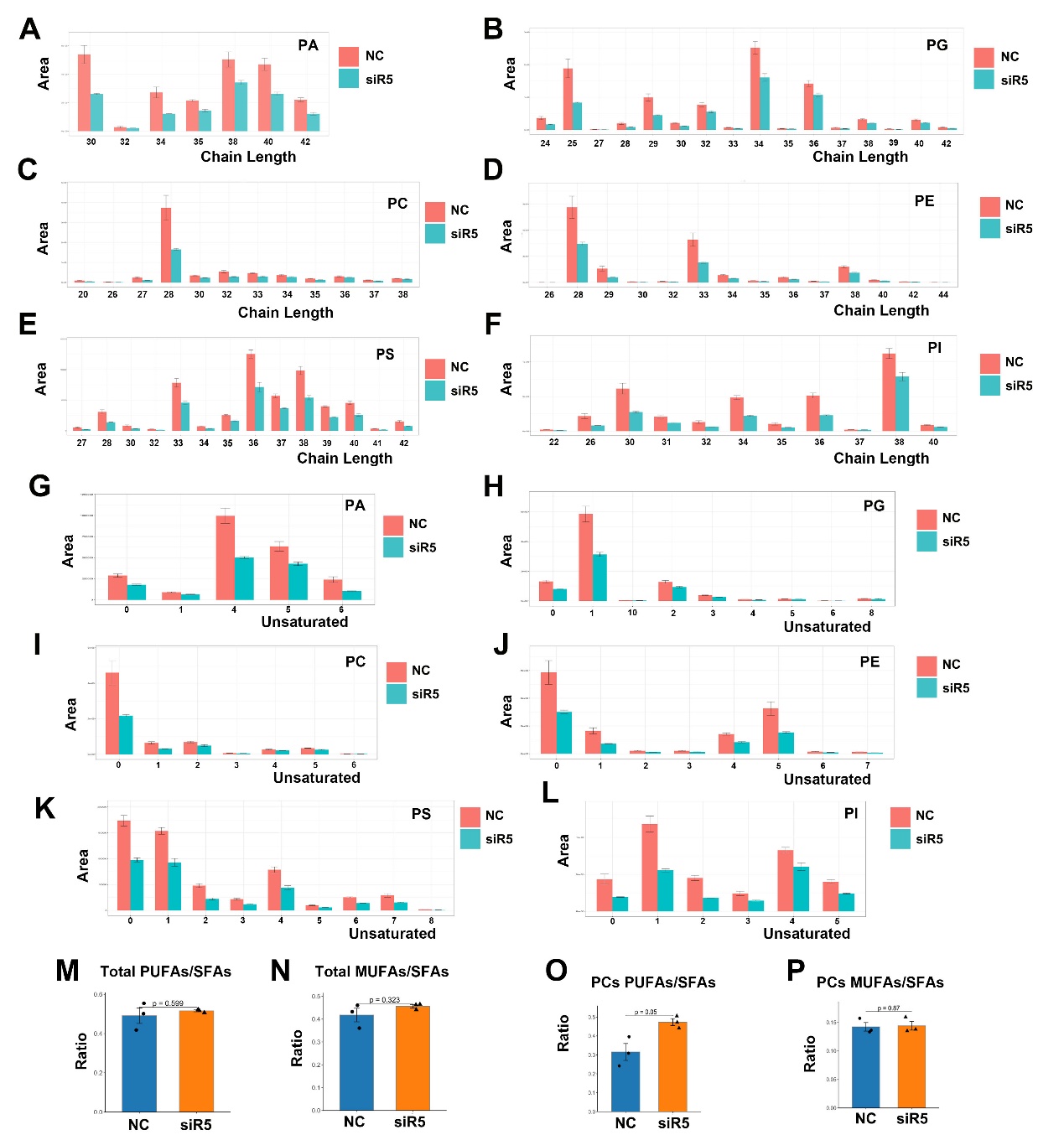
**

**Figure S6. Analysis for the carbon chain length and unsaturation of the lipid compounds.**

**A** Analysis of chain lengths for phosphatidic acid (PA). **B** Analysis of chain lengths for phosphatidylglycerol (PG) **C** Analysis of chain lengths for phosphatidylcholine (PC). **D** Analysis of chain lengths for phosphatidylethanolamine (PE). **E** Analysis of chain lengths for phosphatidylserine (PS). **F** Analysis of chain lengths for phosphatidylinositol (PI). **G** Analysis of unsaturation in PA Lipids. **H** Analysis of unsaturation in PG Lipids. **I** Analysis of unsaturation in PC Lipids. **J** Analysis of unsaturation in PE Lipids. **K** Analysis of unsaturation in PS Lipids. **L** Analysis of unsaturation in PI Lipids. **M** Ratio analysis of total polyunsaturated fatty acids (PUFAs) to saturated fatty acids (SFAs). **N** Ratio analysis of total monounsaturated fatty acids (MUFAs) to SFAs. **O** Ratio analysis of PUFAs to SFAs for PCs lipids. **P** Ratio analysis of MUFAs to SFAs for PCs lipids.
